# An ancient enhancer rapidly evolving in the human lineage promotes neural development and cognitive flexibility

**DOI:** 10.1126/sciadv.adt0534

**Published:** 2025-08-13

**Authors:** Kun Tan, Kendall Higgins, Qing Liu, Miles F. Wilkinson

**Affiliations:** ^1^ Department of Obstetrics, Gynecology, & Reproductive Sciences, University of California San Diego, La Jolla, CA 92093, USA.; ^2^ Institute of Genomic Medicine (IGM), University of California San Diego, La Jolla, CA 92093, USA.

## Abstract

The genetic changes driving the evolution of humans since the human-chimpanzee split have been elusive. Here, we report a promising candidate in a chromosomal region linked with neurological defects—17p13.3. We show that this 442-nucleotide sequence—human-accelerated region (HAR) 123—is a conserved neural enhancer that promotes neural progenitor cell (NPC) formation. While present in all mammals, HAR123 has rapidly evolved since humans diverged from chimpanzees. The human and chimpanzee HAR123 orthologs exhibit subtle differences in their neural developmental effects, and the human HAR123 ortholog uniquely regulates many genes involved in neural differentiation. We identified direct targets of the HAR123 enhancer and showed that *HIC1* acts downstream of HAR123 to promote human NPC formation. HAR123-knockout mice exhibit a defect in cognitive flexibility and a shift in neural-glia ratio in specific regions of the hippocampus. Our study has implications for neurodevelopmental disorders, which are often accompanied by altered neural-glia ratio and have been linked with HARs.

## Teaser

HAR123 is a conserved enhancer rapidly evolving in the human lineage that exerts neural functions.

## INTRODUCTION

The concept that rapidly evolving human sequences are a major force in driving human evolution was first championed in 2006 (*1*, *2*). Two criteria were used to identify such human-accelerated regions (HARs): (i) strong conservation and (ii) rapid sequence changes selectively in the human lineage. The former strongly implies functionality; the latter raises the possibility of positive selection for unique functions in humans (*3*). To date, ~3000 HARs have been defined, with an average length of ~260 nucleotides (nt) (*4*–*6*). The majority of HARs (96%) reside in noncoding regions (*7*). Of these noncoding HARs, many are transcriptional enhancers (*8*–*10*). A recent study provided evidence that almost half of HARs are enhancers (*5*).

Little is known about the biological functions of HARs. Most of what we know comes from studies conducted in mice. Using an overexpression approach in transgenic mice, Boyd *et al.* (*10*) provided evidence that the human version of the frizzled class receptor 8 (FZD8) enhancer, HARE5, increases neural progenitor cell (NPC) proliferation, promotes cortical size, and promotes neuron density. Dutrow *et al.* (*11*) replaced the mouse ortholog of the HAR2 enhancer with its human ortholog and found that this altered the expression of one of its targets, *Gbx2*, in a manner suggesting that HAR2 has a role in the evolution of limb development. Using the same approach, Aldea *et al.* (*12*) showed that *En1* candidate enhancer 18 (ECE18), an enhancer that overlaps with HAR 2XHAR20, regulates the expression of the ENGRAILED-1 transcription factor in a species-biased manner, suggesting a role in the formation of eccrine sweat glands, which are present in higher density in humans than chimpanzees. While these studies have been illuminating, the biological roles of these and other HARs remain poorly understood.

In this study, we report the identification of a HAR that functions in the nervous system. We demonstrate neural roles for this HAR, both in vitro and in vivo, and provide evidence that the human version of this HAR confers several properties—both phenotypic and molecular—that differ from the chimpanzee version, raising the possibility that this HAR has played a role in the evolution of human-specific neural traits.

## RESULTS

### The *SMG6* gene harbors two HARs

**Fig. 1.**
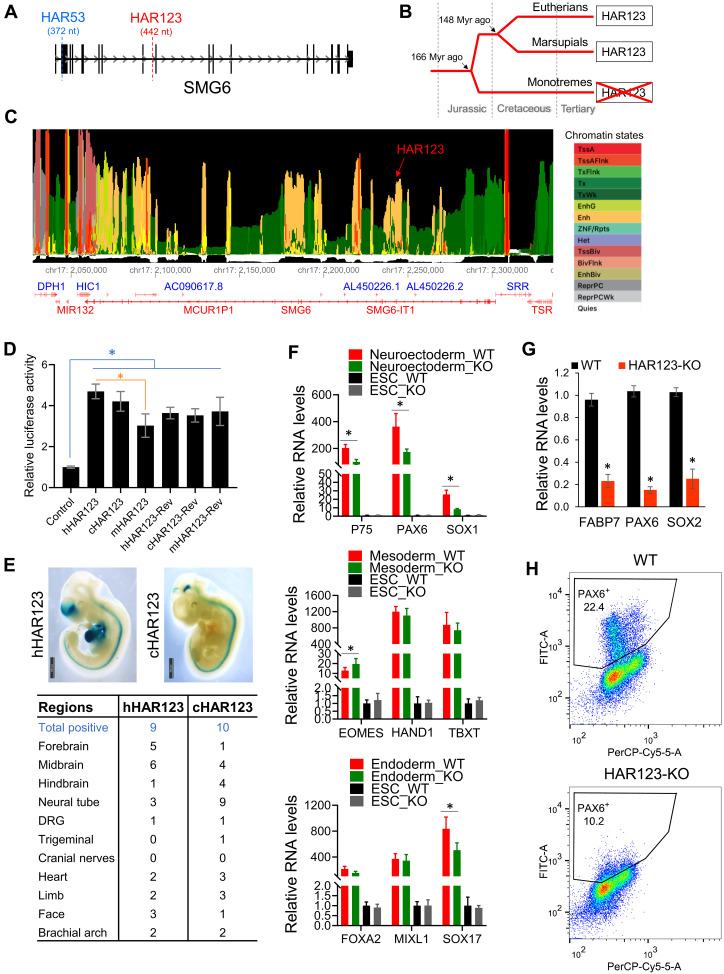
HAR123 is a neural enhancer that promotes human NPC generation. (**A**) Location of HAR53 and HAR123 in the *SMG6* gene. (**B**) HAR123 is present in mammals and marsupials but not in monotremes, based on the University of California Santa Cruz (UCSC) database. (**C**) Human HAR123 has the chromatin marks of an active enhancer, based on the ENCODE map of 15 chromatin states (*17*). The first 7 states listed on the right are associated with active transcription: TssA and TssAFlnk, actively transcribed proximal promoter; TxFlnk, transcribed at the 5′ and 3′ end of genes; Tx and TxWk, strong and weak transcription, respectively; EnhG and Enh, enhancers. The next 7 listed states are associated with repressed transcription: ZNF/Rpts, associated with zinc finger genes; Het, heterochromatin; TssBiv and BivFlnk, bivalent regulatory sites; EnhBiv, bivalent enhancer; ReprPC and ReprPCWk, strong and weak Polycomb-associated repressed sites. Quies, quiescent. (**D**) Luciferase analysis of the pGL3 plasmid (which contains a minimal simian virus 40 promoter upstream of the firefly luciferase gene) harboring human, chimpanzee, or mouse HAR123 in the forward or reverse (Rev) orientation downstream. These six reporter constructs, as well as pGL3 with no HAR123 insert, were transiently cotransfected with a normalization control—a Renilla luciferase reporter plasmid driven by the cytomegalovirus immediate-early promoter (pRL-cmv)—into human embryonic stem cells (hESCs). *n* = 3. *P < 0.05. Data are represented as mean ± SEM. (**E**) Top: Mouse embryos [from embryonic day 11.5 (E11.5)] injected at the one-cell stage with either human (h) or chimpanzee (c) HAR123. Blue denotes β-galactosidase activity. Bottom: Summary of tissues with positive β-galactosidase activity. DRG, dorsal root ganglion. (**F**) Quantitative polymerase chain reaction (qPCR) analysis of wild-type (WT; two clones) and HAR123-KO hESCs (four clones) differentiated into the three primary germ layers using standard protocols (*15*). Shown are well-established markers for the indicated germ layers. *n* = 4. *P < 0.05. Data are represented as mean ± SEM. (**G**) qPCR analysis of NPC markers in WT (two clones) and HAR123-KO hESCs (four clones) differentiated into NPCs using a standard protocol (*20*). *n* = 3. *P < 0.05. Data are represented as mean ± SEM. (**H**) Fluorescence-activated cell sorting (FACS) analysis of the intracellular paired box 6 (PAX6) signal [fluorescein isothiocyanate-area (FITC-A)] in hESCs differentiated as in (G) (detected by permeabilizing the cells before antibody incubation). *n* = 3.

Nonsense-mediated RNA decay (NMD) is a highly conserved and selective RNA turnover pathway that influences both neural development and neuronal function (*13*). Thus, we hypothesized that NMD has undergone changes (quantitative and/or qualitative) during primate evolution to confer human-specific neural traits. One means by which this might have occurred is through human-specific sequence alterations in genes known to encode proteins critical for the NMD pathway. Thus, we screened the following NMD genes—*UPF1*, *UPF2*, *UPF3A*, *UPF3B*, *SMG1*, *SMG5*, *SMG6*, *SMG7*, *SMG8*, *SMG9*, *DHX34*, *NBAS*, *MOV10*, *RUVBL1/2*, *ICE1*, *AKT1*, *CWC22*, *ABCE1*, *CASC3*, *RBM8A*, *MAGOH*, *EIF4A3*, *SF3B1*, and *U2AF1*—for HARs. We screened the 3171 currently defined HARs (*5*) and found that the suppressor with morphogenetic effect on genitalia 6 (*SMG6*) gene harbors two HARs—HAR53 and HAR123 (Fig. 1A). No other NMD genes contain known HARs.

### HAR53 is in the SMG6 coding region

HAR53 initially captured our interest because it is in the coding region of SMG6. Because SMG6 is the endonuclease that degrades NMD target mRNAs (*14*), we assessed whether the amino acid differences between human (h), chimpanzee (c), and mouse (m) HAR53 (fig. S1A) alter NMD magnitude. To address this question, we introduced the nucleotides encoding the amino acids in mHAR53 and cHAR53 into the human *SMG6* gene in human embryonic stem cells (hESCs) using CRISPR-Cas9. The hESCs harboring cHAR53 sequences—cHAR53–knockin (KI) cells—expressed *SMG6* at the same level as wild-type (WT) hESCs (compare hHAR53 with cHAR53-KI in fig. S1B). Consistent with this, NMD magnitude was not significantly different between WT and cHAR53-KI hESCs, as judged by the level of five NMD-target mRNAs (fig. S1B). mHAR-KI hESCs also expressed similar levels of these mRNAs as WT hESCs but did exhibit a statistically significantly different level of *SMG6* and one of five NMD-target mRNAs (fig. S1B).

Next, we asked whether the amino acid differences in mHAR53 and cHAR53 influence primary germ layer formation, based on our previous finding that manipulation of NMD magnitude affects both mesoderm and endoderm formation (*15*). As a positive control, we first tested WT hESCs and found that when they were cultured under conditions to generate endoderm, mesoderm, or neuroectoderm, the well-established markers for these three germ layers were all strongly induced (fig. S1C). We then tested WT, cHAR53-KI, and mHAR53-KI hESCs in parallel and found that they were similarly efficient at forming all three germ layers (fig. S1D). While one of three markers was significantly down-regulated in expression in the cHAR53- and mHAR53-KI cells (relative to WT cells) when induced to form endoderm or neuroectoderm, the altered expression was modest (fig. S1D). With regard to mesoderm, one of three markers was significantly more up-regulated in expression in the cHAR53- and mHAR53-KI cells (relative to WT cells), but this difference was also modest. We also tested these three hESC lines for NPC generation efficiency and found no significant differences (fig. S1, E and F). We conclude that the amino acid differences in the HAR53 region of SMG6 across different mammals do not have a notable effect on NMD magnitude, germ layer formation, or NPC generation.

### HAR123 is a neural enhancer

The other *SMG6* HAR—HAR123—is a 442-nt sequence located in *SMG6* intron 9 (Fig. 1A). HAR123 is an ancient sequence, present in all mammals and marsupials, but not the platypus, a monotreme (Fig. 1B), suggesting that this noncoding sequence arose ~170 million years ago. HAR123 is highly conserved in mammals and marsupials (*16*) but has diverged rapidly since the human-chimpanzee split, with 9-nt differences between hHAR123 and cHAR123.

HAR123 has chromatin marks (*17*) consistent with it being a transcriptional enhancer (Fig. 1C), which we confirmed in transfection experiments with a reporter plasmid containing HAR123 ligated upstream of an enhancer-dependent (minimal) promoter (Fig. 1D). Despite its rapid evolution since the human-chimpanzee split, the chimpanzee version of HAR123 is an equally active transcriptional enhancer as hHAR123 in hESCs (Fig. 1D). The mouse version of HAR123 also exhibited transcriptional enhancer activity (Fig. 1D). All three orthologs exhibited enhancer activity even when inserted in the inverse orientation—the classical definition of an enhancer.

To test whether HAR123 is an enhancer in vivo, we used the “gold-standard” assay for assessing enhancer activity (*18*, *19*). In this in vivo assay, mouse embryos are injected with a plasmid containing the enhancer candidate and an enhancer-dependent (minimal) promoter driving the expression of β-galactosidase (LacZ) (*19*). LacZ expression indicates whether the candidate sequence has enhancer activity, and, if so, what cell lineages it acts in. This in vivo enhancer assay indicated that the human version of HAR123—hHAR123—is a neural enhancer (Fig. 1E), with activity mainly in forebrain (^5^/_9_ LacZ^+^ embryos) and midbrain (^6^/_9_), with only rare activity in hindbrain (^1^/_9_). We also tested cHAR123, which shared with hHAR123 frequent activity in midbrain (^4^/_10_ LacZ^+^ embryos; *P* = 0.22, defined by Fisher’s exact test), but it differed from hHAR123 in also frequently driving expression in hindbrain (^4^/_10_; *P* = 0.15) and rarely driving expression in forebrain (^1^/_10_; *P* = 0.05) (Fig. 1E).

### HAR123 promotes human NPC generation

To determine whether HAR123 has biological functions in human cells, we deleted both HAR123 alleles from hESCs using CRISPR-Cas9. Four independent HAR123–knockout (KO) hESC clones were generated, each with slightly different deletion junctions (fig. S2A). All four clones grew normally, had no obvious morphological differences from control (WT) cells, and exhibited normal expression of pluripotency genes (fig. S2B).

By comparing HAR123-KO with control cells, we asked whether loss of HAR123 causes a defect in forming the three primary germ layers. This analysis revealed that loss of HAR123 specifically perturbed the formation of neuroectoderm, based on reduced expression of three neuroectoderm markers (Fig. 1F). In contrast, HAR123 KO did not notably influence the formation of either endoderm or mesoderm, as only one of three tested markers for each lineage exhibited a significant change in expression, and this change was modest (Fig. 1F). Because neuroectoderm produces NPCs that will generate various types of neurons and glial (nonneuronal) cells, we next asked whether HAR123 is also critical for NPC generation. Using a standard NPC generation protocol (fig. S2C) (*20*), we found that HAR123-KO hESCs exhibit a notable defect in their ability to form NPCs, based on both quantitative polymerase chain reaction (qPCR) and fluorescence-activated cell sorting (FACS) analyses (Fig. 1, G and H).

**Fig. 2.**
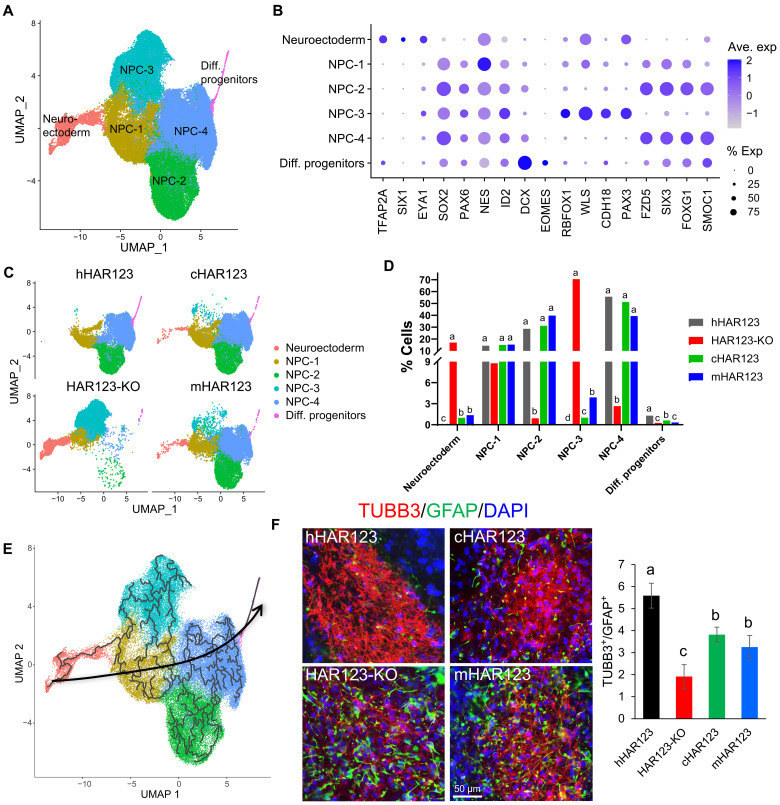
Species-biased effects of HAR123 on human NPC generation. (**A**) scRNA-seq analysis of hESCs differentiated to the rosette stage of NPC generation. hESCs of four genotypes [described in (C)] were assayed independently and analyzed as a group to define the clusters shown in the uniform manifold approximation and projection (UMAP) plot. NPC-1 to -4 are four distinct NPC cell clusters, while the Diff. (differentiating) progenitor cluster has more developmentally advanced cells, based on markers shown in (B) and pseudotime analysis in (E). (**B**) Expression of the gene markers used for annotating the cell types in (A). exp, expression. (**C**) UMAP plots of the same scRNA-seq data as in (A), showing the cell contributions from the four indicated genotypes. (**D**) Quantification and pairwise comparison of the data in (C). Statistical analysis was done using one-way analysis of variance (ANOVA), followed by a Tukey post hoc test. Genotypes with different characters from a given cell cluster (a, b, c, or d) are significantly different in frequency from each other. *P* < 0.05. (**E**) Pseudotime trajectory analysis of the cells in (A). The arrow shows the direction of differentiation. (**F**) Left: Immunofluorescence analysis of differentiating neurons and glial cells from human NPCs of the indicated genotypes (two independent clones of each). NPCs were cultured under differentiation conditions (no fibroblast growth factor 2) for 4 weeks, following a standard neural differentiation protocol (*20*). Tubulin beta 3 class III (TUBB3) and glial fibrillary acidic protein (GFAP) mark neurons and glial cells, respectively. The cells were also stained with 4′,6-diamidino-2-phenylindole (DAPI; blue) to mark nuclei. Scale bar, 50 μm. Two biological replicates were performed. Right: Quantification of neuron/glia ratio of the indicated genotypes. Different letters (a, b, and c) denote statistically significantly different groups (*P* < 0.05).

To examine the role of HAR123 in NPC generation in a more high-resolution manner, we performed single-cell RNA sequencing (scRNA-seq) analysis. For these experiments, we also compared the function of hHAR123 with cHAR123 and mHAR123 by replacing hHAR123 in hESCs with cHAR123 or mHAR123, respectively. Two independent cHAR123-KI and two independent mHAR123-KI hESC clones were generated. We then differentiated these two genotypes of KI-hESCs, along with HAR123-KO and -WT hESCs, into NPCs before the rosette selection step, in parallel. Cells from the two independent clones (from each of the above four genotypes; eight cell clones in total) were pooled together for scRNA-seq analysis. After quality control (fig. S3A), 65,985 cells remained for subsequent analysis. Using a nonlinear dimensionality reduction method, we identified six cell clusters, including four NPC clusters (Fig. 2A). Some of the gene markers used to define these cell clusters are shown in Fig. 2B and fig. S3B. Genes exhibiting enriched expression in each of the six cell clusters are listed in table S1.

Figure 2 (C and D) shows the scRNA-seq–defined cell clusters in the above four genotypes. The data from the KO and WT cells verified that HAR123 KO causes an NPC generation defect, based on two lines of evidence: (i) unlike WT cells, many of the HAR123-KO cells remain as neuroectoderm cells, and (ii) very few HAR123-KO cells advance to form two of the NPC clusters formed by WT cells (NPC-2 and -4). HAR123 loss also caused the formation of an NPC cell cluster not found in WT cells—NPC-3 (Fig. 2, C and D). Using NPC cluster–specific markers (fig. S3C), we validated the accumulation of this NPC-3 cluster in response to HAR123 KO (fig. S3D). To examine the nature of the NPC-3 cluster, we used pseudotime trajectory analysis, which predicts developmental relationships by comparing the transcriptomes of individual cells (*21*). This provided evidence that the NPC-3 cluster is in a developmental state between neuroectoderm and the NPC-2/4 clusters (Fig. 2E). This finding, coupled with accumulation of these NPC-3 cells in the HAR123-KO cells, suggests that these cells are at a transient intermediate stage that requires HAR123 for conversion into NPCs.

To define the molecular effects of HAR123 during NPC generation, we identified genes differentially expressed in each NPC cluster in HAR123-KO versus hHAR123 cells (table S1). These differentially expressed genes (DEGs) are candidates to act downstream of HAR123 in NPC generation, as examined in more detail below. When considering all NPC clusters as a whole, the genes up-regulated upon HAR123 loss are enriched in “cell cycle,” “DNA replication,” and “apoptosis” functions, while down-regulated genes are enriched in “neural development,” “cell migration,” “cell adhesion,” “transcriptional regulation,” and “cell proliferation” functions (fig. S4).

### Species-biased HAR123 effects on NPC generation

To elucidate whether HAR123 has diverged in function in humans versus chimpanzees versus mice, we compared the responses of the cHAR123- and mHAR123-KI hESCs (“cHAR123” and “mHAR123” hESCs, respectively) and the WT (“hHAR123”) and HAR123-KO hESCs. scRNA-seq analysis revealed that both cHAR123 and mHAR123 ESCs formed the NPC-2 and -4 clusters (Fig. 2, C and D). This indicated that the chimpanzee and mouse orthologs of HAR123 rescue the NPC generation defect caused by hHAR123 loss, implying that HAR123 has retained its NPC-promoting function in both primates and rodents, the latter of which is considered to be ~90 million years distant from humans.

However, we found that cHAR123 and mHAR123 differ from hHAR123 in their ability to promote human NPC generation. In contrast to hHAR123 ectodermal cells, which fully differentiate and thus are virtually absent after culture following the NPC differentiation protocol, many cHAR123 and mHAR123 ectodermal cells remain undifferentiated and thus accumulate (Fig. 2, C and D). In addition, many cHAR123 and mHAR123 cells accumulate at the NPC-3 stage, which, as described above, are likely putative transient intermediate cells that do not accumulate when hHAR123 is present (Fig. 2, C and D). Together, these data suggest that the human ortholog of HAR123 subtly differs from its chimpanzee and mouse orthologs in its ability to drive human NPC formation.

To probe into the possibility that the human ortholog of HAR123 has a unique function that differs from the chimpanzee version, we performed reclustering analysis of hHAR123 and cHAR123 cells (fig. S5, A and B). This identified 26 cell clusters, 6 of which significantly differ in their relative proportion in hHAR123 versus cHAR123 cells (fig. S5, B and C). While this differential effect on cell subsets is enticing, its significance is not clear, as cell clusters defined by scRNA-seq are not necessarily functionally unique groups of cells. We also identified genes differentially regulated by hHAR123 versus cHAR123 in different cell subsets defined by scRNA-seq (table S1). Genes more highly expressed in response to hHAR123 than cHAR123 include *BTF3*, *IGFBP5*, and *IGSF1* (fig. S5D), all of which are known to regulate proliferation.

Together, these results suggest that (i) HAR123 is critical for normal human NPC generation, (ii) HAR123 has retained this ability in both primates and rodents, and (iii) the human ortholog of HAR123 confers NPC-related properties and regulates genes that differ from those of human’s closest living relative—the chimpanzee.

### HAR123 influences neuron/glia ratio

Given our discovery that HAR123 promotes NPC generation, we next asked whether HAR123 affects the ratio of the differentiated progeny of NPCs—neurons and glial cells. To address this, we placed the four genotypes of NPCs under standard neural differentiation conditions for 4 weeks. This time point has been shown in past studies to efficiently induce NPC differentiation into mature neurons and glial cells (*20*, *22*–*24*). Comparison of hHAR123 and HAR123-KO NPCs differentiated for 4 weeks revealed that loss of HAR123 caused a statistically significant decrease in neuron/glia ratio, as measured by the TUBB3 and GFAP markers (Fig. 2F). This suggested that HAR123 favors neurons over glial cells. mHAR123 and cHAR123 NPCs exhibited a phenotype similar to hHAR123 NPCs (Fig. 2F), indicating that HAR123 has a conserved role favoring neurons over glial cells. However, hHAR123 and cHAR123 conferred a different neuron/glia ratio (Fig. 2F), suggesting the possibility that HAR123 has undergone a change in its ability to influence neuron/glia ratio since the human-chimpanzee split.

### Species-biased HAR123-regulated genes in ESCs and neuroectoderm

**Fig. 3.**
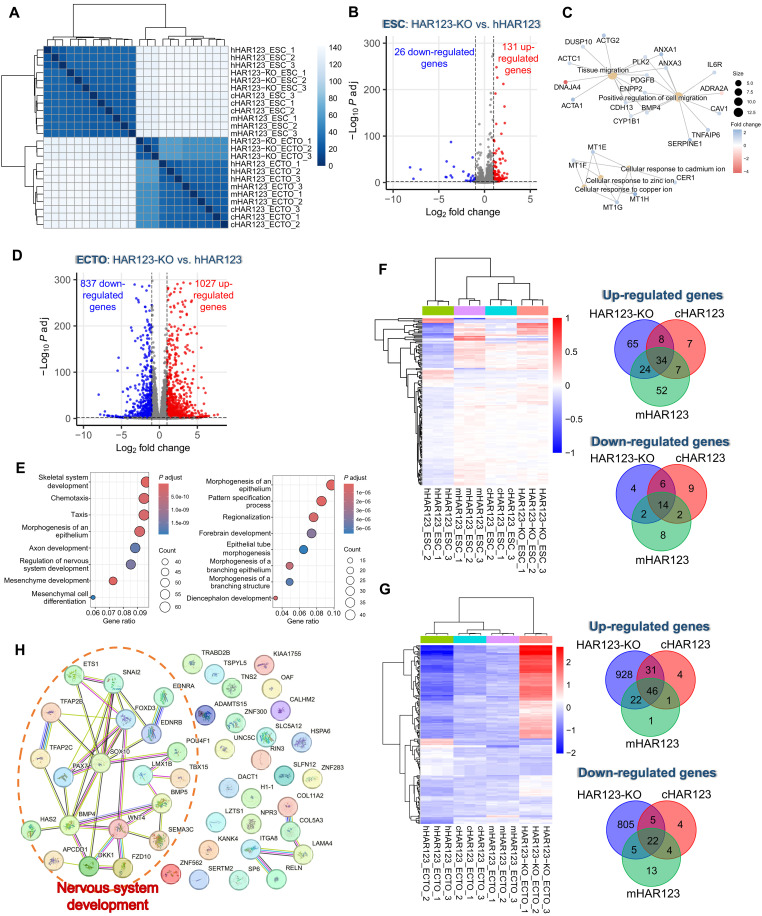
Species-specific gene regulation conferred by HAR123. (**A**) Hierarchical clustering of all RNA-seq samples assayed. ESC, hESCs; ECTO, neuroectoderm. *P* adj, adjusted *P*. (**B**) Volcano plot showing genes differentially expressed between hESCs of the indicated genotypes. *q* < 0.01 and |log_2_ fold change (FC)| > 1. (**C**) Enriched functions and key genes among the DEGs defined in (B), as defined by gene-concept network analysis. (**D**) Volcano plot showing genes differentially expressed between neuroectodermal cells of the indicated genotypes. *q* < 0.01 and |log_2_FC| > 1. (**E**) Dot plots showing the most statistically enriched biological functions encoded by the up-regulated (left) and down-regulated (right) genes defined in (D). (**F**) hESC gene regulation conferred by HAR123 from different species. Left: Heatmap showing the expression pattern of DEGs between the genotypes indicated. Right: Venn plots showing genes differentially expressed between each of the genotypes shown and hHAR123 hESCs. The overlap between all three DEG subsets is genes specifically regulated by hHAR123. (**G**) Neuroectoderm gene regulation conferred by HAR123 from different species. Left: Heatmap showing the expression pattern of DEGs between the genotypes indicated. Right: Venn plots showing genes differentially expressed between each of the genotypes shown and hHAR123 neuroectodermal cells. The overlap between all three DEG subsets is genes specifically regulated by hHAR123. (**H**) Genes uniquely regulated by hHAR123 in neuroectoderm, as defined in (G). The genes are depicted using functional protein association network analysis.

To identify HAR123-regulated genes potentially important for NPC generation, we performed bulk RNA-seq analysis, as this provides >100-fold more reads than scRNA-seq analysis. Because we showed above that HAR123 promotes neuroectoderm formation from ESCs (Fig. 1F) and NPC generation from neuroectoderm (Fig. 1, G and H), we performed RNA-seq analysis at both the ESC and neuroectoderm stages. Two independent clones from each of the four genotypes described above (HAR123-KO, hHAR123, cHAR123, and mHAR123) were used for RNA-seq analysis. Hierarchical clustering showed that biological replicates from each genotype or cell type were clustered together, while the samples with different genotypes were distinct from each other (Fig. 3A).

At the ESC stage, HAR123 KO up- and down-regulated 131 and 26 genes, respectively, as compared to WT (Fig. 3B and table S2). Among the statistically enriched functions associated with these regulated genes is cell migration (Fig. 3C), a process critical for nervous system development. Many more genes were regulated by HAR123 KO at the neuroectoderm stage—1027 and 827 genes were up- and down-regulated, respectively (Fig. 3D and table S2). The regulated genes include those enriched in the following nervous system–related functions: “nervous system development,” “axon development,” “pattern specification process,” “regionalization,” and “forebrain development” (Fig. 3E). The abundance of nervous system developmental genes regulated by HAR123 is consistent with our finding that HAR123 promotes neuroectoderm and NPC generation (Figs. 1 and 2 and fig. S3). The specific genes up- and down-regulated in response to HAR123 loss are candidates to have roles in HAR123’s functions, as evaluated below.

We next asked whether HAR123 has a species-specific effect on gene regulation. We did this by comparing the transcriptomes of hHAR123, cHAR123, mHAR123, and HAR123-KO cells. RNA-seq analysis revealed that, at the ESC stage, 34 and 14 genes are uniquely down-regulated and up-regulated, respectively, by hHAR123 (not cHAR123 or mHAR123) (Fig. 3F and fig. S6A). At the neuroectoderm stage, 46 and 22 genes are uniquely down- and up-regulated, respectively, by hHAR123 (Fig. 3G). We verified the regulation of a random selection of these DEGs by qPCR analysis (fig. S6, B and C). Almost one-half of the genes specifically regulated by the human ortholog of HAR123, not the chimpanzee or mouse orthologs, encode proteins involved in nervous system development (Fig. 3H). One of the genes down-regulated by hHAR123, not cHAR123, is *FOXD3* (Fig. 3H), which encodes a member of the forkhead family of transcription factors required for maintenance of the multipotent mammalian neural crest (*25*). Another gene in this class is *BMP4* (Fig. 3H), which encodes a key signaling protein involved in maintaining an undifferentiated pool of neural stem cells and neural progenitors (*26*). The finding that the human version of HAR123 uniquely regulates a cadre of genes involved in nervous system development supports the possibility that HAR123 has evolved to alter one or more neurodevelopmental traits in the human lineage since the human-chimpanzee split.

### Identification of direct target genes of HAR123

**Fig. 4.**
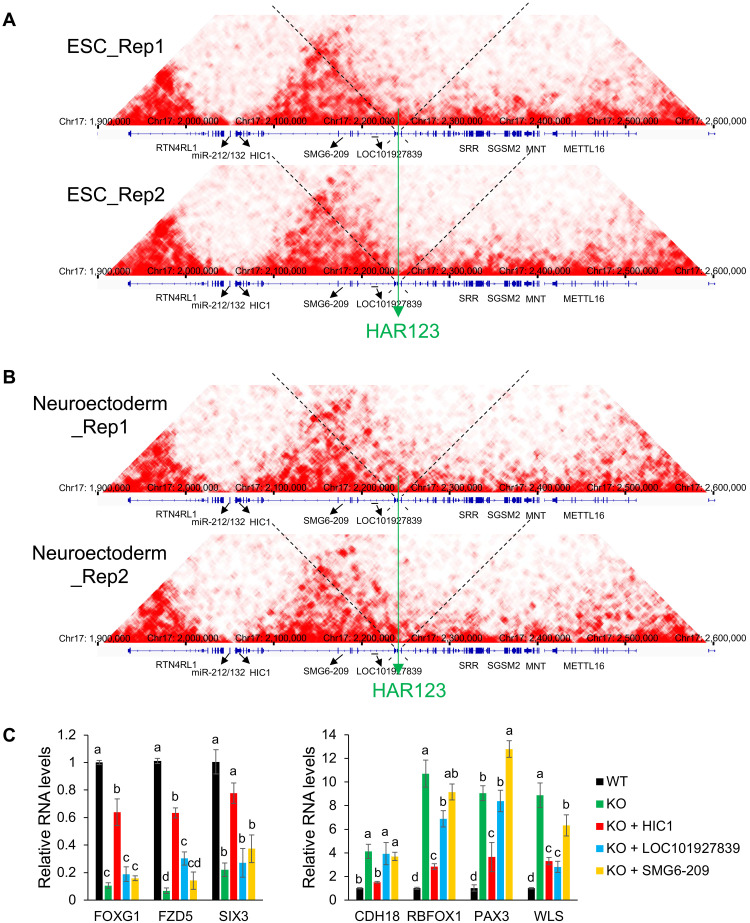
A HAR123-*HIC1* circuit drives human NPC generation. (**A**) Hi-C analysis of hESCs, showing the long-range chromatin interactions between HAR123 and specific sites. Rep, replicate. (**B**) Hi-C analysis of neuroectodermal cells showing the long-range chromatin interactions between HAR123 and specific sites. (**C**) qPCR analysis of hHAR123 (WT) and HAR123-KO hESC cells cultured following a well-established NPC generation protocol (*20*). At the ESC stage, the cells were transduced with lentiviral viruses expressing the indicated transcripts. The data show that forced expression of *HIC1* rescues NPC generation, as shown by the up-regulation of the three NPC-2 markers (FOXG1, FZD5, and SIX3) and down-regulation of the four NPC-3 markers (CDH18, RBFOX1, PAX3, and WLS) (see fig. S3C). Statistical analysis was done using one-way ANOVA, followed by a Tukey post hoc test. Different letters denote statistically significantly different groups (*P* < 0.05). *n* = 3 (from four independent HAR123-KO clones). Data are represented as mean ± SEM.

Given that HAR123 is a transcriptional enhancer (Fig. 1, C to E), we hypothesized that it transcriptionally activates specific genes to drive NPC generation. As a first step to identify HAR123-target genes, we performed high-throughput chromosome conformation capture (Hi-C) analysis on hESCs and neuroectodermal cells (Fig. 4, A and B). Statistically significant HAR123-interacting sites in both cells were identified only in the topologically associating domain harboring HAR123 on chromosome 17 (table S3), analogous with the cis-specificity of many other enhancers (*27*). The most prominent (and frequent) HAR123-interacting site is in the neighboring gene, *HIC1* (Fig. 4, A and B, and table S3), which encodes a transcriptional repressor (*28*). There are also statistically significant interactions (false discovery rate < 0.05) in the gene that houses HAR123—the NMD gene *SMG6* (Fig. 4, A and B, and table S3). However, there are no statistically significant HAR123-interacting sites within 5 kb of the *SMG6* promoter that drives expression of the full-length SMG6 protein, suggesting that HAR123 does not act as an enhancer to promote NMD in ESCs or neuroectoderm. In support, the level of full-length *SMG6* mRNA was not significantly reduced in HAR123-KO hESCs or neuroectoderm relative to WT hESCs or neuroectoderm, respectively (table S2). Consistent with this, SMG6 splicing and NMD magnitude were not significantly affected in these HAR123-KO hESCs as compared to WT hESCs (fig. S6, D and E).

To define functional HAR123-enhancer targets among these HAR123-interacting regions, we made use of our RNA-seq datasets, as described above (table S2). This revealed that *HIC1* mRNA is down-regulated in response to loss of HAR123 (verified by qPCR analysis; fig. S7A). Coupled with the visually notable interaction of *HIC1* with HAR123 (Fig. 4, A and B), these data indicate that *HIC1* is a high-confidence HAR123 target. We also examined whether the large (>200 kb) *SMG6* locus, which encodes >15 annotated transcripts, is regulated by HAR123. Several of the promoters driving these transcripts were within 5 kb of HAR123-interacting sites, but we only identified three *SMG6*-locus transcripts that were detectably expressed in ESCs and neuroectoderm (verified by qPCR analysis; fig. S7A). Two of these transcripts—*SMG6-209* and the long noncoding RNA antisense transcript *LOC101927839*—are down-regulated in response to HAR123 loss (verified by qPCR analysis; fig. S7A), indicating that these are high-confidence HAR123-enhancer targets. HAR123 also interacts with the tandem microRNA cluster miR-212/132 (table S3), raising the possibility that HAR123 also acts as an enhancer to stimulate the expression of these miRNAs. However, neither miR-212 nor miR-132 expression is down-regulated in HAR123-KO ESC and neuroectoderm (fig. S7B). Thus, these miRNAs are unlikely to be HAR123-enhancer targets in these cells. We conclude that *HIC1* and two *SMG6*-locus internal transcripts (*LOC101927839* and SMG6-209) are targets of the HAR123 enhancer. All three of these transcripts are regulated by HAR123 in neuroectoderm, not ESCs [as shown by both RNA-seq analysis (table S2) and qPCR analysis (fig. S7A)], consistent with our in vivo data showing that HAR123 is a neural enhancer (Fig. 1E).

### Identification of a HAR123-*HIC1* circuit that drives human NPC generation

To test whether any of the above three HAR123-enhancer targets function downstream of HAR123 to drive NPC generation, we performed a rescue experiment. We asked whether forced expression of these transcripts (to a level similar to WT levels; fig. S7C) rescued the NPC generation defect of HAR123-KO hESCs. The NPC subset–specific markers we used for this analysis were defined by scRNA-seq (fig. S3C). We found that forced expression of *HIC1*, but not *SMG6-209* or *LOC101927839*, rescued the NPC generation defect (Fig. 4C). Together with our finding that *HIC1* is HAR123’s direct target, these results suggest the existence of a HAR123-*HIC1* circuit that promotes human NPC generation. The human ortholog of HAR123 may be unique in its ability to stimulate *HIC1* expression, as human neuroectodermal cells that instead harbor the chimpanzee or mouse orthologs of HAR123 express low levels of *HIC1* mRNA similar to the level in HAR123-KO neuroectoderm (fig. S7D).

### HAR123 is critical for cognitive flexibility in vivo

**Fig. 5.**
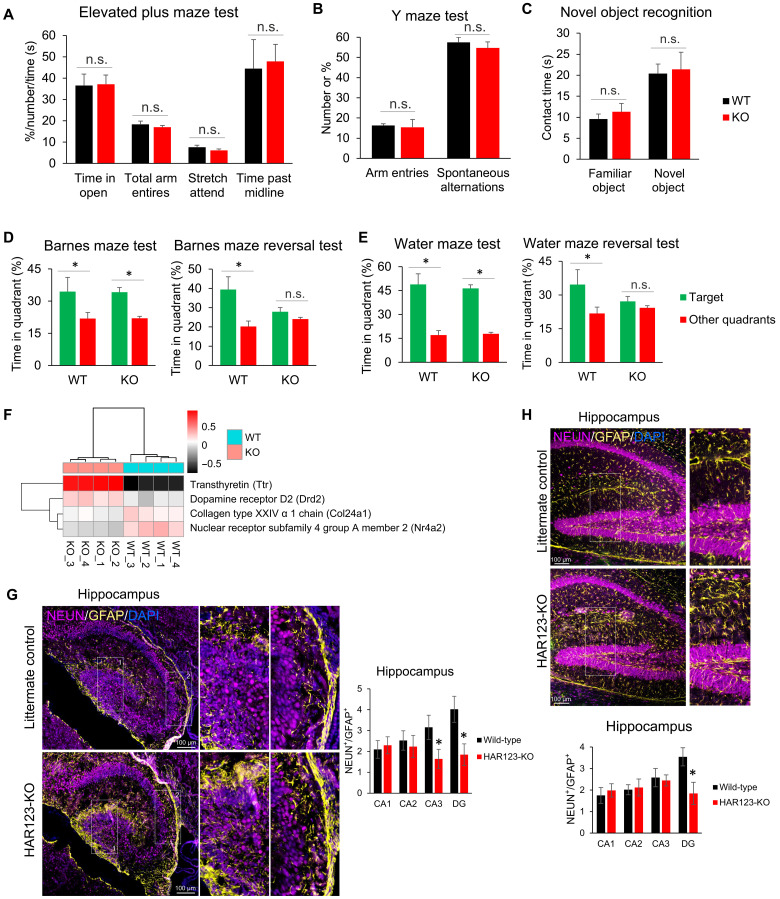
HAR123-KO mice exhibit defects in the hippocampus and reversal learning. (**A**) to (**E**) show the results of behavioral assays performed on adult HAR123-KO and control (WT) littermate mice (*n* = 16 mice for each genotype). *P < 0.05. Data are represented as mean ± SEM. (**F**) Heatmap showing that genes encoding proteins known to be associated with cognitive flexibility are dysregulated in the frontal cortex of HAR123-KO mice as compared to control (WT) littermate mice, based on RNA-seq analysis (see fig. S8C). (**G**) Left: Immunofluorescence analysis of the hippocampus from postnatal–day 7 (P7) mice of the indicated genotypes. Neuronal nuclei (NEUN) labels neurons, GFAP labels glial cells, and DAPI labels nuclei. The number of NEUN^+^ and GFAP^+^ cells was counted manually. Scale bars, 100 μm. Right: Quantification based on analysis of brain sections from three individual mice from each genotype (two sagittal sections per animal). *P < 0.05. (**H**) Top: Immunofluorescence analysis of the hippocampus from P35 mice of the indicated genotypes, performed as in (G). Bottom: Quantification based on analysis of brain sections from two individual mice from each genotype (three sagittal sections per animal).

To determine whether HAR123 functions in vivo, we deleted this enhancer in mice using CRISPR-Cas9. We found that HAR123-KO mice are viable, fertile, and have normal litter size. To determine whether loss of HAR123 causes behavioral defects, we performed a series of behavioral assays, which showed that the HAR123-KO mice exhibit no statistically significant differences (compared to littermate WT mice) in the following behavioral tests: rotarod, locomotor activity, object recognition, optomotor Y maze, elevated plus maze (EPM), standard Barnes maze, and standard Morris maze (Fig. 5, A to C, fig. S8, A and B, and table S4). While the HAR123-KO mice exhibited normal learning and memory to an initial cue, they failed to correctly respond to a second cue, as judged by two independent “reversal learning” tests (Fig. 5, D and E, fig. S8C, and table S4) (*29*, *30*). In these tests, the visual cue that permits the mice to find the target is moved from the original “learned location.” Together, these results suggest that while HAR123-KO mice have a normal ability to initially learn and remember, they are deficient in their ability to learn and/or remember altered information; i.e., they have a defect in cognitive flexibility.

### HAR123-regulated genes in the frontal cortex

To define the potential mechanisms underlying the ability of HAR123 to function in cognitive flexibility, we used RNA-seq analysis to identify HAR123-regulated genes in the adult frontal cortex (fig. S8D), as this region has previously been shown to be involved in cognitive flexibility (*31*–*34*). RNA-seq analysis identified a small cadre of dysregulated genes in the HAR123-KO frontal cortex: 35 up- and 12 down-regulated genes (fig. S8E and table S5). Statistically enriched biological functions for these DEGs include responses to “endogenous stimulus” and “hormone,” as well as “mesangial cell–matrix adhesion” and “cell chemotaxis” (fig. S8F). Several of the dysregulated genes—including *Ttr*, *Nr4a2*, *Col24a1*, and *Drd2* (Fig. 5F)—have been previously shown to encode proteins involved in cognitive flexibility (*35*–*38*).

Given that we found that the mouse ortholog of HAR123 can partially substitute for the human ortholog in promoting hESC differentiation (Fig. 2), this predicts that these two orthologs of HAR123 regulate some of the same genes. When we compared HAR123-KO mice frontal cortex DEGs (table S5) with HAR123-KO human neuroectodermal cell DEGs (table S2), we found that *KLK6*, *ASS1*, *PADI2*, and *CLIC6* are regulated by HAR123 in both scenarios. *KLK6*, *ASS1*, and *PADI2* are known to be involved in neural system development (*39*–*41*). We note that this overlap of mHAR123- and hHAR123-regulated genes is likely to be an underestimate, as adult frontal cortex cells and developing neuroectodermal cells are quite different.

### Evidence that HAR123 influences neuron/glia ratio in vivo

Because we found that loss of HAR123 in differentiating human NPCs caused a decrease in neuron/glia ratio (Fig. 2F), we examined whether HAR123-KO had the same effect in mice in vivo. We assessed this ratio in the hippocampus and frontal cortex regions, as both have been implicated as having a role in cognitive flexibility (*31*, *33*, *34*, *42*–*45*). For comparison, we also analyzed the cerebellum. We chose to initially analyze postnatal–day 7 (P7) mice, as this stage represents a critical period of brain development, characterized by active neurogenesis and gliogenesis (*46*), allowing us to capture dynamic changes in neuronal and glial populations. Using NEUN and GFAP to detect neurons and glial cells, respectively (*47*–*49*), we found that the CA3 and dentate gyrus (DG) regions of the P7 hippocampus exhibited a significantly lower neuron/glia ratio in HAR123-KO mice as compared to littermate control mice (Fig. 5G). In contrast, neuron/glia ratio was not significantly reduced in the CA1 or CA2 regions of the hippocampus, or in the cerebellum, of HAR123-KO mice (fig. S9A). Because P7 is an early developmental stage when both neurons and glial cells are still developing, we also assessed neuron/glia ratio at a later postnatal stage—P35. This revealed a statistically significant decrease in neuron/glia ratio in the DG region (Fig. 5H). Thus, neuron/glia ratio was reduced in the DG region of HAR123-KO mice at both early (P7) and late (P35) postnatal time points. In contrast, no statistically significant change in neuron/glia ratio was observed in the other P35 brain regions we tested in HAR123-KO mice (although there was a trend toward reduced neuron/glia ratio in frontal cortex; fig. S9B). Together, these results suggest that HAR123 influences neuron/glia ratio in specific hippocampus regions at both the early and late postnatal stages.

## DISCUSSION

The mechanism by which unique traits were acquired in the human lineage since the human-chimpanzee split has intrigued people since even before the theory of evolution was first advanced by Charles Darwin. Since then, several non–mutually exclusive genetic mechanisms have been proposed to be involved, including human-specific functions provided by recently formed human-lineage genes (*50*) and human-specific amino acid substitutions in particular proteins (*51*). In this communication, we focus on a third mechanism—human-specific regulatory circuits. There has been considerable interest in this mechanism, particularly with respect to the role of HARs in human brain evolution. In support of HARs conferring human-specific neural traits, HAR-associated genes have been shown to be enriched in synaptic functions and regulatory networks in the prefrontal cortex (*3*, *52*). In addition, HARs have been shown to be linked to human-specific neurodevelopmental disorders, including autism (*8*, *53*) and schizophrenia (*52*).

While progress has been made in understanding HARs, their biological functions have remained poorly understood. For example, studies that have “knocked in” hHARs into mice have not identified phenotypic defects attributable to such HARs (*11*, *12*). These deficiencies in the field cannot be overstated, as it is critical to define the functions of HARs in vivo before one can address whether they confer human-specific traits. Moreover, while there have been a plethora of studies defining HAR enhancers (*3*), few direct targets of these HAR enhancers have been definitively defined, even in studies that have performed in-depth studies on putative HAR targets (*10*–*12*).

Here, we provide several lines of evidence that HAR123 acts in the nervous system. First, we demonstrated through both in vivo and in vitro experiments that HAR123 is a conserved neural enhancer. Second, we also showed that HAR123 drives the formation of human NPCs. This function is conserved, as our knock-in studies show that both the chimpanzee and mouse orthologs of HAR123 share this function with hHAR123. However, it remains to be determined precisely how HAR123 influences NPC generation; e.g., it may act by promoting NPC generation, NPC proliferation, and/or accelerating the timing of NPC generation. Future investigations will also be required to determine whether HAR123 promotes NPC generation in vivo. Third, we generated HAR123-KO mice and found that they have a specific defect in “relearning” (but not “initial learning”), suggesting that HAR123 functions in cognitive flexibility. This role of HAR123 is intriguing, as cognitive flexibility may be more advanced in humans than nonhuman primates (*54*). While it remains to be determined whether HAR123 is involved in behavior in humans, it is known that microdeletions that include HAR123 (as well as neighboring genes) are associated with behavioral defects (*55*–*57*). Last, we found that HAR123 influences the ratio of neurons and glial cells produced from NPCs both in vitro and in vivo. This is intriguing given that a delicate balance between neurons and glial cells is crucial for brain development, synaptic function, and neuroplasticity (*58*, *59*). The specific brain region of HAR123-KO mice that we found had an altered neuron/glia ratio is the hippocampus. Given that the hippocampus is known to play a role in cognitive flexibility (*43*, *45*), it is tempting to speculate that HAR123 might promote cognitive flexibility through its ability to influence neuron/glia ratio in the hippocampus. HAR123 may also play a role in neural diseases, as neuron/glia ratio imbalances are linked to neurodevelopmental disorders, including autism and schizophrenia, as well as neurodegenerative diseases such as Alzheimer’s and Parkinson’s (*60*).

Our data suggest that the human ortholog of HAR123 has several properties that differ from its chimpanzee ortholog. First, hHAR123 and cHAR123 differ in their ability to drive the formation of human NPC subsets, as assessed by scRNA-seq analysis. Second, hHAR123 and cHAR123 differ in their molecular effects. We identified genes regulated specifically by hHAR123, not cHAR123, in ESCs, neuroectoderm, and NPC subsets, as defined at both the single-cell– and/or deep-sequencing level. Third, we obtained evidence that hHAR123 and cHAR123 differ in their spatial activity as neural enhancers, with the former acting preferentially in forebrain and the latter acting preferentially in hindbrain. However, a caveat is that our evidence for this spatial selectivity comes from a standard enhancer activity assay involving randomly integrated plasmid DNA in mouse embryos (*19*). We also note that we did not observe a statistically significant difference in enhancer activity of hHAR123 and cHAR123 in hESCs. Further analysis will be required to determine whether differences in enhancer activity emerge between hHAR123 and cHAR123 as pluripotent cells develop into more developmentally advanced cell types. Last, we identified a molecular circuit specifically involving the human ortholog HAR123 that drives NPC generation. In particular, we identified a target gene of hHAR123, *HIC1*, which our rescue experiments indicate acts downstream of hHAR123 to drive human NPC generation. We found that only the human ortholog of HAR123, not cHAR123 or mHAR123, drives human *HIC1* expression, raising the possibility that this is a human-specific molecular circuit driving NPC generation. However, these data were obtained from human cells, and thus, we cannot rule out that cHAR123 regulates *HIC1* in chimpanzee cells. We also note that it is likely that HAR123 also acts through gene targets in addition to *HIC1* to drive NPC generation.

The finding that the human ortholog of HAR123 differs in some of its properties from the cHAR123 ortholog raises the possibility that HAR123 has a role in the originally hypothesized function of HARs—to confer human-specific traits (*1*). However, this remains to be determined. For example, while we demonstrated that HAR123 orthologs from different species differentially influence NPC generation, this conclusion was drawn from comparing the effects of HAR123 orthologs knocked into hESCs. It is possible that cHAR123 has different effects than hHAR123 in human neural cells because of coevolution of cognate factors that act in a species-specific manner with HAR123 since the human-chimpanzee split.

The extreme conservation of HARs across diverse species strongly suggests that many HARs in addition to HAR123 influence biological functions. To date, however, the evidence for this has been circumstantial. In part, this is likely due to the fact that the particular HARs that have been studied in depth (*5*, *10*–*12*) are enhancers, which typically act redundantly, and thus, it is often difficult to elucidate their function by a loss-of-function approach. For example, Aldea *et al.* (*12*) found that deletion of the eccrine gland–promoting ECE18 enhancer (which overlaps with three HARs collectively named 2XHAR20) failed to cause detectable eccrine sweat gland defects.

Our discovery that HAR123 acts through a neighboring gene, *HIC1*, to drive NPC generation is intriguing. Hypermethylated in cancer 1 (HIC1) is a transcriptional repressor that has been reported to function in several developmental processes, including craniofacial development (*61*), a process linked to neurodevelopmental disorders. Our data support a model in which HIC1 represses the transcription of genes that maintain neuroectoderm in an undifferentiated state. When HAR123 is knocked out, this reduces HIC1 expression and thus allows these neuroectoderm-promoting genes to remain expressed, thereby suppressing the formation of NPCs.

HAR123 and *HIC1* are in the middle of a highly unstable chromosomal region—17p13.3—that is responsible for several rare neurodevelopmental disorders, including Miller-Dieker lissencephaly syndrome and isolated lissencephaly sequence (*55*). *HIC1* has been suggested to be a causal gene in these syndromes (*56*); our findings implicate HAR123 as also being a candidate to have a role in these neurodevelopmental disorders. Both HAR123 and *HIC1* deletions perfectly correlate with brain white matter abnormalities in a set of patients with 17p13.3 microdeletion (*57*). It will be intriguing in the future to determine the precise roles of HARs in both neural diseases and human nervous system traits.

## MATERIALS AND METHODS

### Mice

This study was carried out in strict accordance with the guidelines of the Institutional Animal Care and Use Committee (IACUC) at the University of California, San Diego. The protocol was approved by the IACUC at the University of California, San Diego (permit number: S09160). All mice were housed under a 12-hour–light:12-hour–dark cycle and provided with food and water ad libitum. All mouse strains used for analysis were backcrossed to C57BL/6J for at least eight passages. For mating, males (aged 8 to 16 weeks) and females (8 to 16 weeks) were used.

### Cell culture

hESCs (WA09) were maintained in StemFlex medium (Gibco) on plates coated with hESC-qualified Matrigel (Corning). For expansion, colonies were split (1:10 ratio) after Accutase (Gibco) treatment. Mycoplasma testing was carried out routinely using the MycoAlert mycoplasma detection kit (Lonza).

As described previously (*15*), for neuroectodermal differentiation, hESCs were seeded as single cells at 6 × 10^4^ cells/cm^2^. Once confluent, the hESCs were cultured in KnockOut Dulbecco’s modified Eagle’s medium (DMEM; Gibco) containing 10% KnockOut serum replacement (Gibco) and supplemented with Noggin (500 ng/ml; R&D Systems) and SB431542 (10 μM; Selleck Chemicals) for 5 days. For mesoderm differentiation, hESCs were seeded as single cells at 1 × 10^4^ cells/cm^2^. After 24 hours, the media were replaced with STEMdiff mesoderm induction medium (STEMCELL Technologies) for 4 days. For endoderm differentiation, hESCs were seeded as single cells at 1 × 10^4^ cells/cm^2^. After 24 hours, the media were replaced with DMEM F12/RPMI (1:1) medium (Gibco) supplemented with NaHCO_3_ (2.5 μg/ml; Sigma-Aldrich), 1% GlutaMAX (Gibco), glucose (5.5 mM; Sigma-Aldrich), 0.1% fatty acid free bovine serum albumin (BSA) (Sigma-Aldrich), and insulin-transferrin-selenium-ethanolamine supplement (ITS-X; 1:100; Gibco) supplemented with wingless-type MMTV integration site family, member 3A (WNT3A; 20 ng/ml), bone morphogenetic protein 4 (BMP4) (10 ng/ml; R&D Systems), and activin A (AA; 100 ng/ml) for 1 day, followed by incubation for 3 days with the same media containing AA (100 ng/ml) only.

As described previously (*20*), for generating NPCs, hESCs were seeded as single cells at 6 × 10^4^ cells/cm^2^. Once 80% confluent, the hESCs were cultured in DMEM/F12 (Gibco) supplemented with N2 (1:100; Gemini), 5 μM Rho-associated protein kinase (ROCK) inhibitor, 1 μM dorsomorphin, and 10 μM SB431542 (STEMCELL Technologies) for 2 days. Colonies were then cut into a “big chess pattern” and cultured in DMEM/F12 (Gibco) supplemented with N2 (1:100; Gibco), 1 μM dorsomorphin, and 10 μM SB431542 for 5 days under rotation (95 rpm) inside a 37°C incubator. Embryoid bodies were then replated on plates coated with hESC-qualified Matrigel (Corning) in DMEM/F12 media supplemented with N2 (1:200; Gemini) and Gem21 (1:100; Gemini) for 7 days. Neural rosettes were picked and dissociated with Accutase (Gibco) and plated on plates coated with poly-ornithine/laminin in DMEM/F12 media supplemented with N2 (1:200; Gemini), Gem21 (1:100; Gemini), and basic fibroblast growth factor (bFGF) (20 ng/ml; Sigma-Aldrich). Homogeneous populations of NPCs were achieved after two to three passages with Accutase under the same conditions. For neural differentiation, NPCs were grown to ~80% confluence and then cultured in the same media without bFGF. ROCK inhibitor (5 μm) was added for the first 2 days, and then the cells were allowed to differentiate for 4 weeks, with a medium change twice per week.

Human embryonic kidney (HEK) 293T cells obtained from the American Type Culture Collection were cultured in DMEM (Gibco) with 10% fetal bovine serum (Gibco) and antibiotics.

### Transfection and viral transduction

HEK293T cells were transfected with the Lipofectamine 2000 reagent (Invitrogen). hESCs were transfected with Lipofectamine Stem Transfection Reagent (Invitrogen). Transfection was performed following the manufacturer’s instructions. For lentiviral transduction, ~1 infectious unit of lentivirus per cell was added with polybrene (5 μg/ml).

For reporter analysis, HEK293T cells and hESCs were transfected with the HAR123/pGL3 constructs described and harvested 1 day posttransfection for luciferase activity analysis. Luminescence was measured using the Dual-Luciferase Reporter assay system (Promega) following the manufacturer’s instructions. The pRL-cmv (Renilla) vector was cotransfected in these experiments as an internal control for normalization.

### CRISPR-mediated genome editing

To delete HAR123 in hESCs, the CRISPOR (*62*) online tool was used to design guide RNAs (gRNAs) (#1, TGTTCAGCAGTAACCACGGG; #2, GCACGACTTCTGGGCACCAG), and DNAs corresponding to these were cloned into the PX459 backbone (*63*) separately. To generate cHAR123- and mHAR123-KI hESCs, the donor containing chimpanzee or mouse HAR123 plus human homology arms was cloned into the Puc19 vector. The desired sequences were verified by Sanger sequencing. gRNAs and/or donor vectors were delivered using the nucleofector system (Lonza) following the manufacturer’s instructions. Once positive clones were generated, they were validated as having a normal karyotype using Illumina Infinium BeadChips.

To delete HAR123 in mice, gRNAs (#1, AATTATGTTGTGCTAACCCA; #2, CACCACTGTTTAGGATCCAG) were designed using the CRISPOR online tool and synthesized by integrated DNA technologies (IDT). A mix of Alt-R S.p. HiFi Cas9 Nuclease V3 (final concentration of 20 ng/ul; IDT) and single gRNA (50 ng/ul) in injection buffer [10 mM tris (pH 7.5) and 0.1 mM EDTA] was injected into the pronucleus of C57BL/6J embryos. The injected zygotes were cultured in M16 medium with amino acids at 37°C with 5% CO_2_ for ~2 hours, and then the zygotes were transferred into the uterus of pseudopregnant CD1 females, the surrogate mothers.

To generate cHAR53- and mHAR53-KI hESCs, gRNAs (#1, TTAGCAAAGAATCCGCCAGT; #2, AGGCACCACACGCCGATTGT) were designed using the CRISPOR online tool, and the DNAs corresponding to these were cloned into the PX459 backbone separately. The donor containing chimpanzee or mouse HAR53 plus human homology arms was cloned into the Puc19 vector. The desired sequences were verified by Sanger sequencing. gRNAs and/or donor vectors were delivered using the nucleofector system (Lonza) following the manufacturer’s instructions. Once positive clones were generated, they were validated as having a normal karyotype using Illumina Infinium BeadChips.

### Mouse transgenic enhancer assay

Human and chimpanzee HAR123 sequences were inserted into the Hsp68-LacZ-Gateway vector (Addgene), upstream of a minimal Hsp68 promoter. The inserted sequences were verified by Sanger sequencing. The constructs were linearized by restriction digest with aspartate-proline-alanine I (ApaI) and injected into the pronuclei of C57BL/6J embryos. The injected zygotes were cultured in M16 medium with amino acids at 37°C under 5% CO_2_ for ~2 hours, and then the zygotes were transferred into the uterus of pseudopregnant CD1 females. Embryos were collected at embryonic day 11.5 (E11.5), incubated for 30 min with 4% paraformaldehyde at 4°C, and then washed three times with wash buffer [2 mM MgCl_2_, 0.01% deoxycholate, 0.02% NP-40, and 100 mM phosphate buffer (pH 7.3)]. Then, embryos were stained for 24 hours at room temperature with freshly made staining solution [X-galactosidase (0.8 mg/ml), 4 mM potassium ferrocyanide, 4 mM potassium ferricyanide, and 20 mM tris (pH 7.5) in wash buffer], followed by three washes in phosphate-buffered saline (PBS) and postfixed in 4% paraformaldehyde. All the above chemicals were purchased from Sigma-Aldrich. The images were viewed using a Leica S6D microscope.

### Reverse transcription qPCR analysis

Total cellular RNA was isolated using the Direct-zol RNA MiniPrep Plus kit (Zymo Research), following the manufacturer’s instructions. As described previously (*64*), reverse transcription (RT)–PCR analysis was performed using 1 mg of total cellular RNA using the iScript cDNA synthesis kit (Bio-Rad), followed by PCR amplification using SYBR Green (Bio-Rad) and the ΔΔCt method (using the ribosomal gene, *RPL19*, for normalization). The primers are listed in table S6.

### Immunofluorescence analysis

Differentiating NPCs were fixed in 4% paraformaldehyde for 0.5 hour on ice and blocked with 0.25% Triton X-100 (Sigma-Aldrich) and 5% donkey serum (Sigma-Aldrich) in PBS at room temperature for 1 hour. Cells were then incubated overnight with the primary antibody [TUBB3 (1:100; Proteintech #66375-1-Ig) or GFAP (1:100; Proteintech #16825-1-AP)] at 4°C and subsequently with secondary antisera (Alexa Fluor 488 and 555) for 1 hour at room temperature. The nuclei were counterstained with 4′,6-diamidino-2-phenylindole (DAPI; Vector Laboratories). The images were viewed using a Leica DMI4000 B fluorescence microscope.

### Flow cytometry

Cells were harvested, washed with PBS, fixed, and permeabilized using the True-Nuclear Transcription Factor Buffer Set (BioLegend). Intracellular PAX6 expression was determined using a rabbit antihuman PAX6 antibody (Proteintech #12323-1-AP; 1:100 dilution) incubated for 30 min on ice. After washing with staining buffer, the cells were incubated with an Alexa Fluor 488 antirabbit secondary antibody for 20 min on ice, resuspended in staining buffer, and analyzed by FACS. Forward scatter gating was set to exclude small debris and doublets. As a negative control, only the secondary antibody was used.

### RNA-seq analysis

RNA-seq was performed as described previously (*65*–*70*). Total RNA was extracted using the RNeasy Plus Mini kit (QIAGEN), following the manufacturer’s protocol. Ribosomal RNAs were removed using NEBNext rRNA Depletion Kit v2 (New England Biolabs). This enriched mRNA was then used to make the library with the NEBNext Ultra II Directional RNA Library Prep kit (New England Biolabs), as per the manufacturer’s instructions. Libraries were sequenced (paired-end reads) with an Illumina NovaSeq 6000 platform for 100 cycles at the University of California San Diego (UCSD) Institute for Genomic Medicine (IGM) core. Reads were filtered for quality and aligned with STAR (2.5.2b) against GRCh38 (Ensembl version 106) or GRCm38 (Ensembl version 96). The exon counts were aggregated for each gene to build a read count table using the Subread function featureCounts. DEGs were defined using the DESeq2 program with the following thresholds: |Log_2_ fold change (FC)| > 1 and *q* < 0.01. The R package program, pheatmap, was used for clustering and generating heatmap plots. The R package program, clusterProfiler, was used for gene functional annotation.

### scRNA-seq analysis

hESCs with the genotypes described (two independent clones of each genotype) were differentiated into NPCs using a standard NPC generation protocol (*20*), as described in the “Cell culture” section above. Just before the rosette selection step, the cells were collected for scRNA-seq analysis, which was performed as previously described (*65*, *71*–*73*). Briefly, the cells were dissociated into a single-cell suspension using Accutase (Gibco), dead cells were removed using the ClioCell Dead Cell Removal kit (Amsbio) following the manufacturer’s instructions, viable cells were washed once in PBS, resuspended in 0.04% BSA in PBS, pooled by group, and used for loading on the 10x Chromium chip. To reduce potential batch effects, four groups were processed simultaneously. Cell capturing and library preparation were carried out as per kit instructions (Chromium Next GEM Single Cell 3′ HT v3.1), with 20,000 cells (10,000 cells per loading well) targeted for capture per sample. After cDNA synthesis and library amplification, the resultant libraries were size selected, pooled, and sequenced using the 2 × 100 paired-end sequencing protocol on an Illumina NovaSeq 6000 instrument at the IGM core.

Demultiplexed raw sequencing reads were processed and mapped to the human genome (GRCh38) using CellRanger software (v7.0.1) with default parameters. Filtered count matrices for each library were tagged with a library batch ID and combined across independent experiments using the Seurat package (version 4.4.0) (*74*) in R. To check the quality of the single-cell data and remove any multiplets, we performed Seurat-based filtering of cells based on more stringent criteria than used previously (*71*): number of detected features (nFeature_RNA) per cell (2000 to 10,000) and percentage of mitochondrial genes expressed (<0.1). Gene expression values were log normalized. Batch correction was performed using the “JackStraw” functions in the Seurat package. To identify cell clusters, we used uniform manifold approximation and projection (UMAP). The “FindMarkers” function (a Wilcoxon rank sum test) was used to determine differential gene expression between clusters (set at minimum expression in 25% of cells). Gene Ontology analysis (DAVID v6.8) was done using top DEGs (positively) with a *P*-adjusted cutoff of 0.01. Single-cell pseudotime trajectories were constructed with the Monocle3 package (v1.3.4) (*21*).

### Hi-C analysis

H9 hESCs and neuroectodermal cells were cross-linked with 1% formaldehyde for 10 min at room temperature and incubated with 3 M tris (pH 7.5) for 5 min at room temperature and then on ice for 15 min to quench the formaldehyde. The cells were centrifuged for 10 min at 800*g* at 4°C, the pellet was resuspended in cold 1× PBS, and the cell density was determined. Indexed libraries were prepared using 2 × 10^6^ cells, with EpiTect Hi-C (QIAGEN) according to the manufacturer’s instructions. The pooled library was sequenced using the 2 × 150 paired-end sequencing protocol on an Illumina NovaSeq 6000 instrument.

Paired-end reads were aligned separately to the human genome [version hg38; University of California Santa Cruz(UCSC)] using bowtie2, with low mapping quality filtered, and paired and PCR duplicates removed. The output binary alignment map (BAM) files were transformed into tag directories for further analysis using the program HOMER. We used the contact matrix at 1- and 10-kb resolution. Topologically-associated domains were identified, and significant interactions (*P* < 1 × 10^–10^, *z*-score > 10) were identified using HOMER.

### Behavioral tests

All behavioral tests were performed at the Animal Models Core Facility at the Scripps Research Institute. Comparisons were made between HAR123-KO mice and WT littermates (4 months old). Sixteen individuals (eight males and eight females) per group were used for these tests. Analysis of variance (ANOVA) was used for the statistical analyses of behavioral results, followed by post hoc Student’s *t* tests, as appropriate, to calculate the *P* values.

#### EPM test

The EPM apparatus consisted of a 5 cm–by–5 cm central square connected to two opposite open arms and two opposite closed arms. Each arm is 30 cm long and 5 cm wide. The closed arms has 15-cm-high walls, while the open arms has 3-mm-high ledges. The runway is placed on top of a 30-cm-high stand. The runway floors were made of matte gray acrylic, and all other surfaces were made of clear acrylic. The mouse was placed in the central square of the apparatus, facing a closed arm, and allowed to explore freely for 5 min. The apparatus was wiped with 70% EtOH inbetween mice trials. The test was recorded by a camera mounted above the EPM and connected to a computer. The distance traveled, number of entries, and time spent in each area of the EPM were calculated by ANY-maze (Stoelting Co.). The total distance traveled was used as an index of locomotor activity, and the time spent in the open arms was used as an index of anxiety-like behavior. The apparatus design (open arms with ledges and closed arms with transparent walls) is meant to encourage exploration of the open arms and facilitate the detection of an anxiogenic-like effect of alcohol withdrawal.

#### Locomotor activity test

Locomotor activity was measured in polycarbonate cages (42 cm by 22 cm by 20 cm) placed into frames (25.5 cm by 47 cm) mounted with two levels of photocell beams 2 and 7 cm above the bottom of the cage (San Diego Instruments, San Diego, CA). These two sets of beams allowed for the recording of both horizontal (locomotion) and vertical (rearing) behaviors. A thin layer of bedding material was applied to the bottom of the cage. Mice were tested for 120 min, and data were collected in 5-min intervals.

#### Optomotor test

The optomotor allows for assessment of visual ability and consists of a stationary elevated platform surrounded by a drum with black-and-white striped walls. Each mouse was placed on the platform to habituate for 1 min, and then the drum was rotated at 2 rpm in one direction for 1 min, stopped for 30 s, and then rotated in the other direction for 1 min. The number of head tracks (15° movements at speed of drum) was recorded.

#### Y maze test

Spontaneous alternation behavior—a measure of spatial working memory, exploratory behavior, and responsiveness to environmental change—was tested using a Y maze with 34 cm–by–8 cm–by–14 cm arms. Each mouse was evaluated in a single 5-min trial, and spontaneous alternations, sets of three unique arm choices, were recorded.

#### Open field test

The apparatus used is a white PLEXIGLAS square (50 cm by 50 cm by 22 cm), with an open field illuminated to 400 lux in the center. Each mouse was placed in the center of the field, and several behavioral parameters (distance traveled, velocity, and center time) were recorded during a 60-min observation period and analyzed using Noldus EthoVision XT software.

#### Object recognition test

To evaluate short-term memory, mice were individually habituated to an open field for 5 min and tested with two identical objects placed in the field using two 5-min trials separated by a 1-min interval. After another 1-min delay, one of the familiar objects was replaced with an unfamiliar one, and object contact time was recorded to assess recognition memory.

#### Rotarod test

Using a Smartrod apparatus (Omnitech Electronics, Columbus, OH, USA), a protocol was applied whereby the rod begins in a stationary state and then starts to rotate with a constant acceleration of 20 rpm. When the mice were incapable of staying on the moving rod, they fell past photobeams to the bedding-covered floor below. The speed of the rod at the time of fall was recorded by a computer. The mice were tested nine times on the first day (three sets of three) with 1 min between each test within a set and ~1 hour between each set. On the next day, the mice were retested to examine the retention of the motor learning developed on the previous day.

#### Barnes maze test and reversal test

The Barnes maze test is used to assess spatial learning and memory in rodents. Mice learn to find an escape tunnel among 20 possibilities below an elevated, brightly lit, and noisy platform, using cues placed around the room. Spatial learning and memory were assessed across trials and then directly analyzed on the final probe trial in which the tunnel was removed, and the time spent in each quadrant was determined; the percent time spent in the target quadrant (the one originally containing the escape box) was compared with the average percent time in the other three quadrants. For the reversal test, the probe was rotated 180° from the original position.

#### Water maze test and reversal test

The water maze test is used to assess spatial learning and memory in rodents. Mice are placed into a circular tub filled with opaque water, and they learn over repeated trials to locate a hidden platform onto which they can sit and escape from the swimming. Each mouse underwent two trials per day for 4 days, with a fixed platform location and a random start position. After being released into the water, each mouse was allowed to swim until the platform was found or 90 s had elapsed, at which point the experimenter gently guided the mouse to the platform. A probe trial was given after the completion of training (day 5), in which the platform was removed from the water maze and the mouse was allowed to swim freely for 90 s. The amount of time spent in each quadrant, the number of times a mouse entered a quadrant, and the number of times the mouse crossed the platform location (annulus crossings) were recorded using Noldus EthoVision software. For the reversal test, the probe was rotated 180° from the original position.

### Statistical analysis

Graphs were generated using GraphPad Prism 10 Project or Microsoft Office Excel. All numerical data are presented as the means ± SEM or SD as needed. Differences between groups were compared by *t* test or one-way ANOVA. The following symbols were used to denote statistical significance: *P < 0.05; n.s., not significant. Statistical analysis for omics data was described in each section above.
