# Supplementary material for: An ancient enhancer rapidly evolving in the human lineage promotes neural development and cognitive flexibility
Source: Sci Adv. 2025 Aug 13;11(33):eadt0534. doi: 10.1126/sciadv.adt0534 (PMC12346275; doi:10.1126/sciadv.adt0534)
Supplement: Supplementary file 1 — Figs. S1 to S9 Tables S5 and S6 Legends for tables S1 to S4 [file sciadv.adt0534_sm.pdf]

Science Advances
AAAS

Supplementary Materials for

An ancient enhancer rapidly evolving in the human lineage promotes neural development and cognitive flexibility

Kun Tan *et al.*

Corresponding author: Kun Tan, kutan@health.ucsd.edu; Miles F. Wilkinson, mfwilkinson@health.ucsd.edu

Sci. Adv. **11**, eadt0534 (2025)
DOI: 10.1126/sciadv.adt0534

The PDF file includes:

Figs. S1 to S9
Tables S5 and S6
Legends for tables S1 to S4

Other Supplementary Material for this manuscript includes the following:

Tables S1 to S4

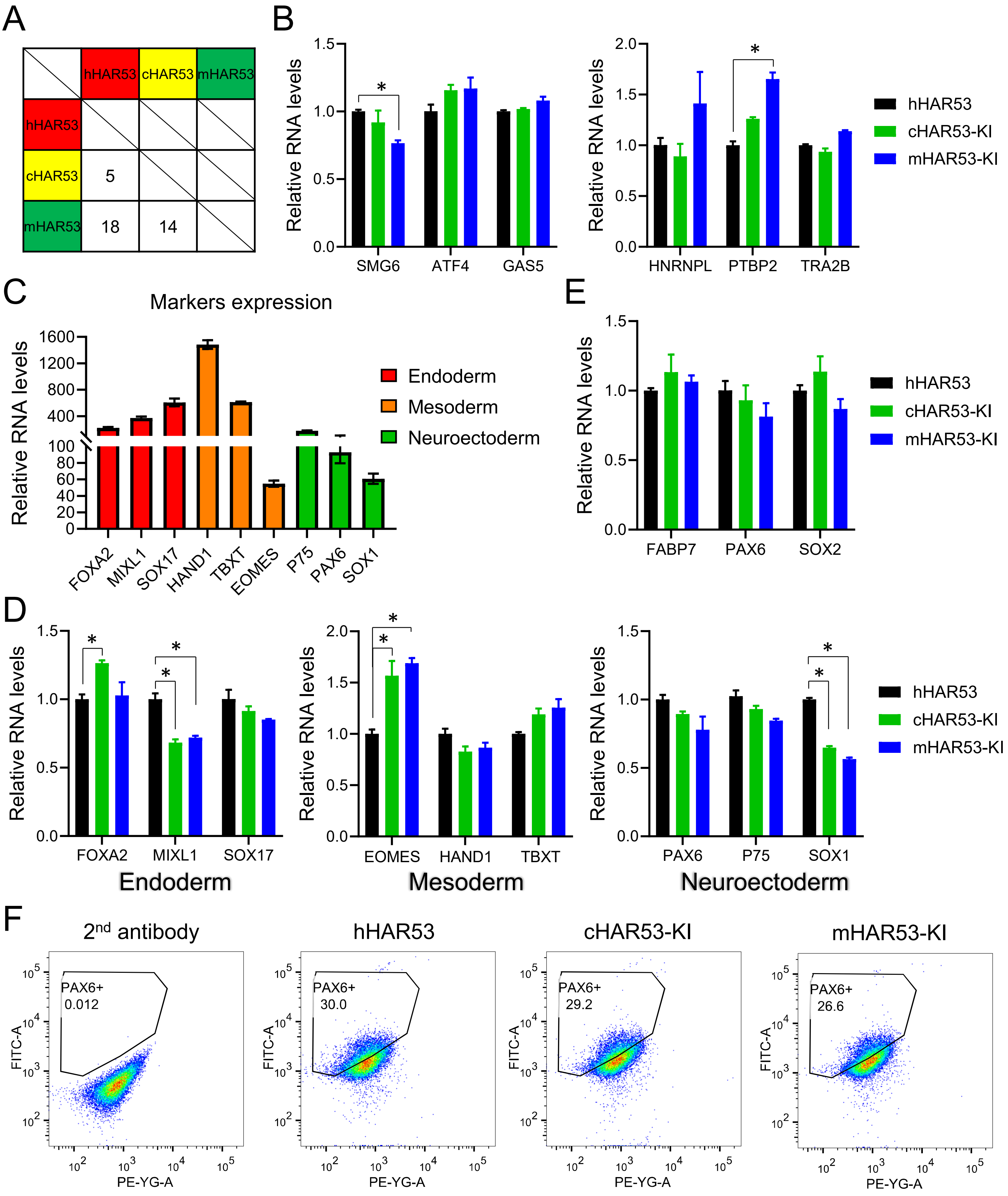
A
hHAR53
cHAR53
mHAR53
5
18
14
B
Relative RNA levels
SMG6
ATF4
GAS5
HNRNPL
PTBP2
TRA2B
hHAR53
cHAR53-KI
mHAR53-KI
C
Markers expression
Relative RNA levels
Endoderm
Mesoderm
Neuroectoderm
FOXA2
MIXL1
SOX17
HAND1
TBXT
EOMES
P75
PAX6
SOX1
E
FABP7
PAX6
SOX2
D
FOXA2
MIXL1
SOX17
Endoderm
EOMES
HAND1
TBXT
Mesoderm
PAX6
P75
SOX1
Neuroectoderm
F
2nd antibody
hHAR53
cHAR53-KI
mHAR53-KI
PAX6+
0.012
30.0
29.2
26.6
FITC-A
PE-YG-A

fig. S1. The sequence differences between human (h), chimpanzee (c), and mouse (m) HAR53 have little or no influence on NMD activity in hESCs, primary germ layer formation, or NPC generation. (**A**) Amino acids differences between HAR53 from the indicated species. HAR53 is SMG6 amino acids 238 to 362. (**B**) qPCR analysis of hESCs with the indicated genotypes. Left, *SMG6* mRNA and NMD target (*ATF4* and *GAS5*) mRNAs. Right, the ratio of NMD-targeted isoform to non-NMD targeted isoform of the indicated mRNAs, a robust measure of NMD magnitude. All values are relative to WT (hHAR53) cells, which is given a value of "1." n = 3. Data are represented as mean ± SEM. (**C**) qPCR analysis of the indicated markers in WT H9 hESCs differentiated into the 3 primary germ layers, using a standard protocol (*15*). The expression levels shown are relative to undifferentiated hESCs, which is a given a value of "1." n = 3. Data are represented as mean ± SEM. (**D**) qPCR analysis of the indicated markers (see panel C for lineage) in hESCs of the indicated genotype differentiated into the 3 primary germ layers. n = 3. Data are represented as mean ± SEM. (**E**) qPCR analysis of NPC markers in hESCs of the indicated genotype cultured following a standard NPC generation protocol (*20*). n = 3. *, $p < 0.05$. Data are represented as mean ± SEM. (**F**) FACS analysis of the NPC marker, PAX6, in hESCs with the indicated genotypes that were cultured under standard conditions to differentiate them into NPCs (*20*). PAX6 is a nuclear protein, so it was detected by permeabilizing the cells before antibody incubation. The PAX6 antibody is FITC-A labeled.

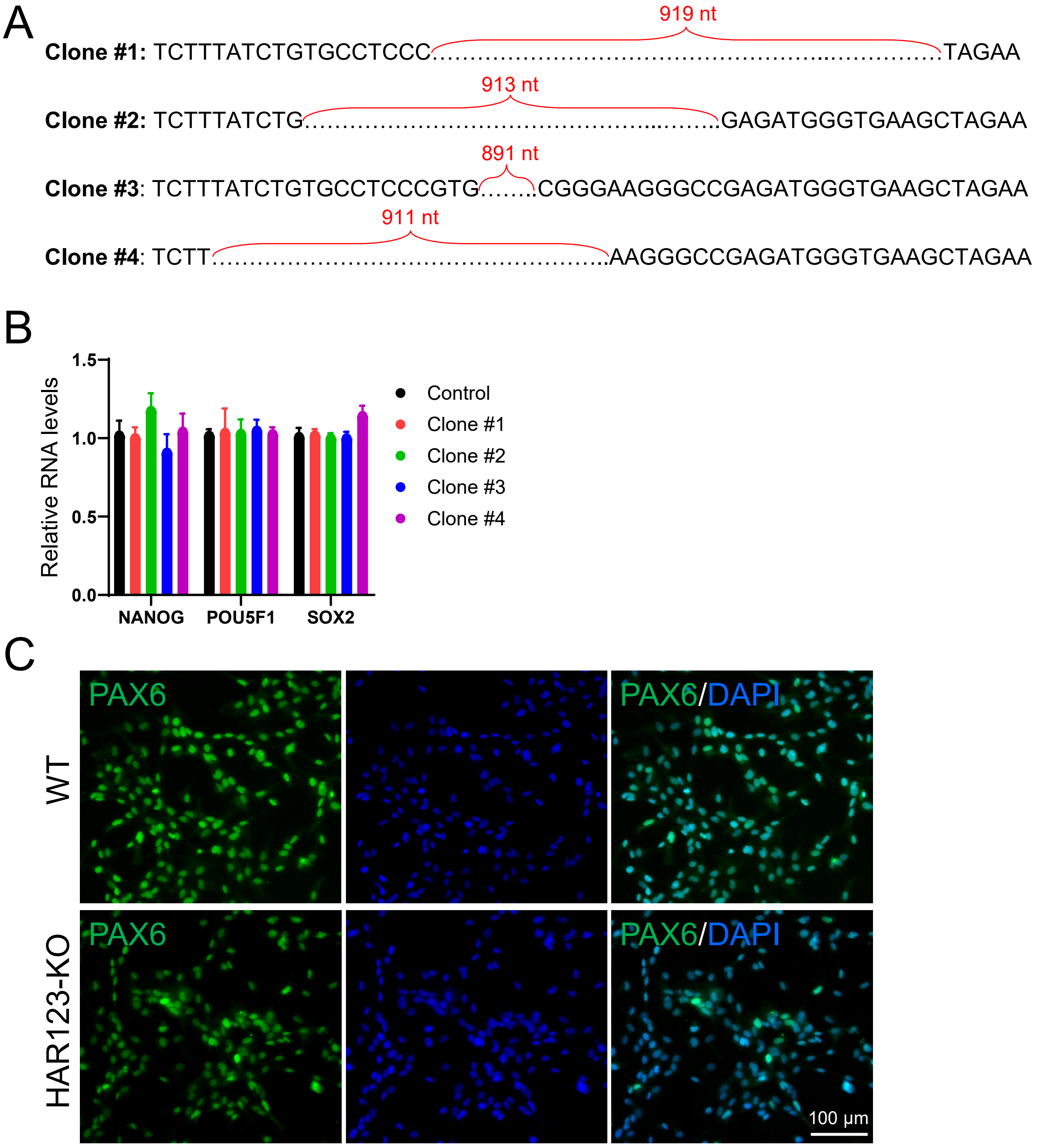

fig. S2. KO of the HAR123 enhancer in hESCs. (**A**) The deletion boundary of the HAR123 region in 4 independent HAR123-KO hESC clones generated using CRISPR. (**B**) qPCR analysis of the expression of the indicated pluripotency markers in H9 control and the 4 HAR123-KO clones. n = 2. Data are represented as mean ± SEM. (**C**) Immunofluorescence analysis of NPCs generated from WT and HAR-123 KO hESCs following a standard protocol (*20*). PAX6 marks NPCs. The cells were also stained with DAPI (blue) to mark nuclei. Scale bar, 100 µm. n = 2.

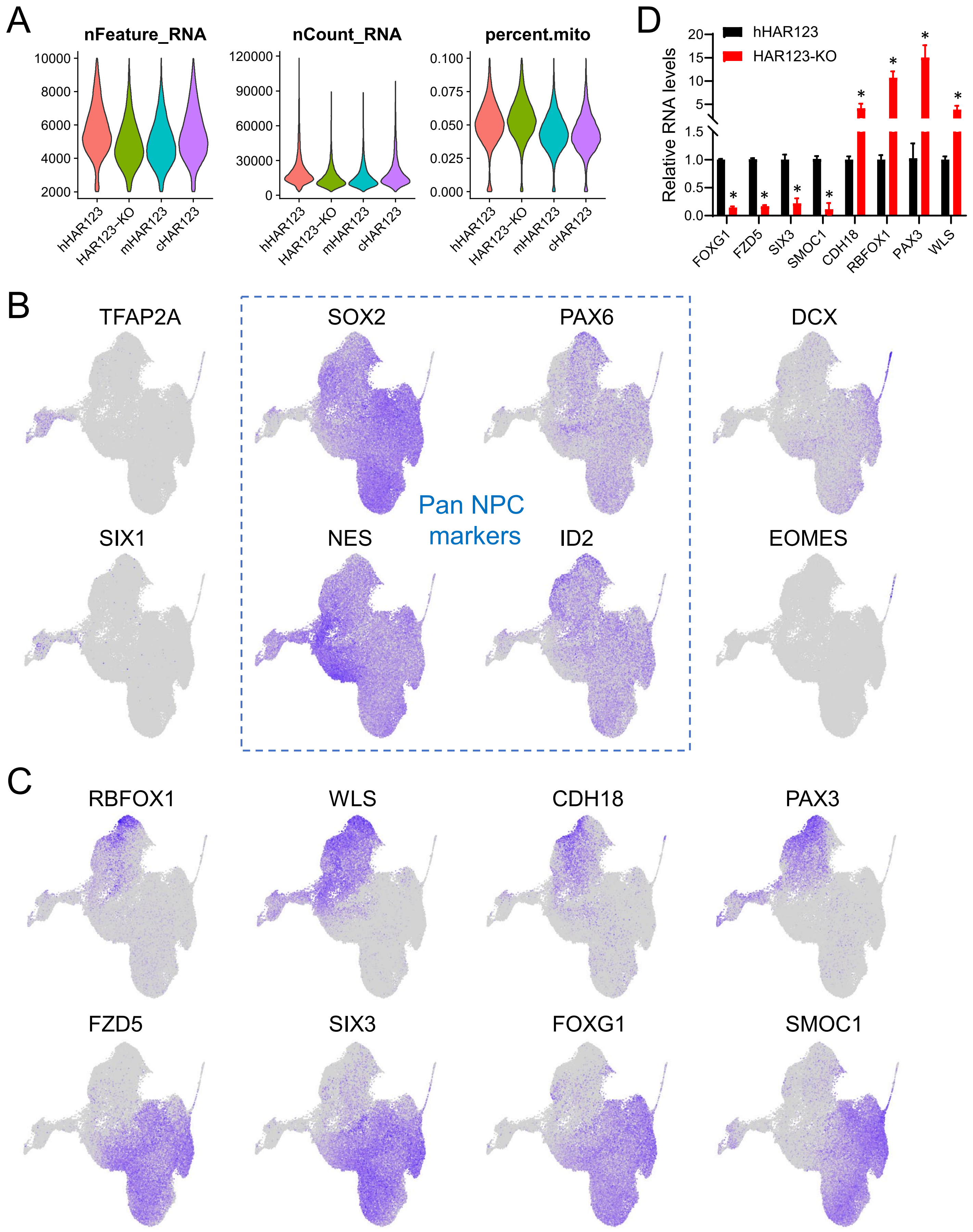
A
nFeature_RNA
nCount_RNA
percent.mito
hHAR123
HAR123-KO
mHAR123
cHAR123
D
hHAR123
HAR123-KO
Relative RNA levels
FOXG1
FZD5
SIX3
SMOC1
CDH18
RBFOX1
PAX3
WLS
B
TFAP2A
SOX2
PAX6
DCX
Pan NPC markers
SIX1
NES
ID2
EOMES
C
RBFOX1
WLS
CDH18
PAX3
FZD5
SIX3
FOXG1
SMOC1

fig. S3. Identification of cell-cluster markers and their usage to demonstrate that the NPC-3 subset is dramatically increased when HAR123 is deleted. (**A**) Violin plots of the indicated parameters from the scRNAseq data in Fig. 2A. hESCs of the indicated genotypes were cultured following a standard NPC generation protocol (*20*). (**B**) UMAP plots of markers for neuroectoderm (left), pan-NPC (middle, boxed), and differentiating progenitors (right). See Fig. 2A for identity of the cell clusters. (**C**) UMAP plots of markers for NPC-3 (top) and NPC-2/4 (bottom). (**D**) qPCR analysis of hESCs of the indicated genotype cultured as in Fig. 2A, using the markers from panel C. n = 3 (from 4 independent HAR123-KO clones). *, $p < 0.05$. Data are represented as mean ± SEM.

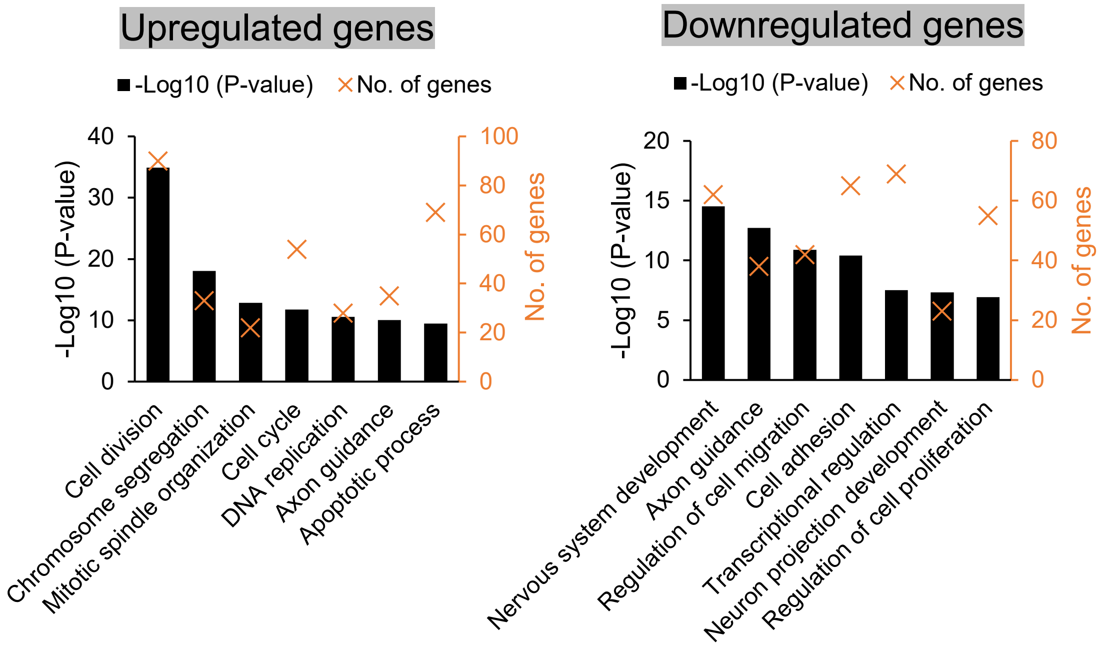

fig. S4. Categories of HAR123-regulated genes in NPCs. The most statistically enriched biological functions encoded by genes up- and down-regulated in HAR123-KO vs. hHAR123 (WT) NPC clusters, when aggregated as a group, as determined by scRNAseq analysis (Fig. 2). The Xs indicate the number of HAR123-regulated genes for a given GO term.

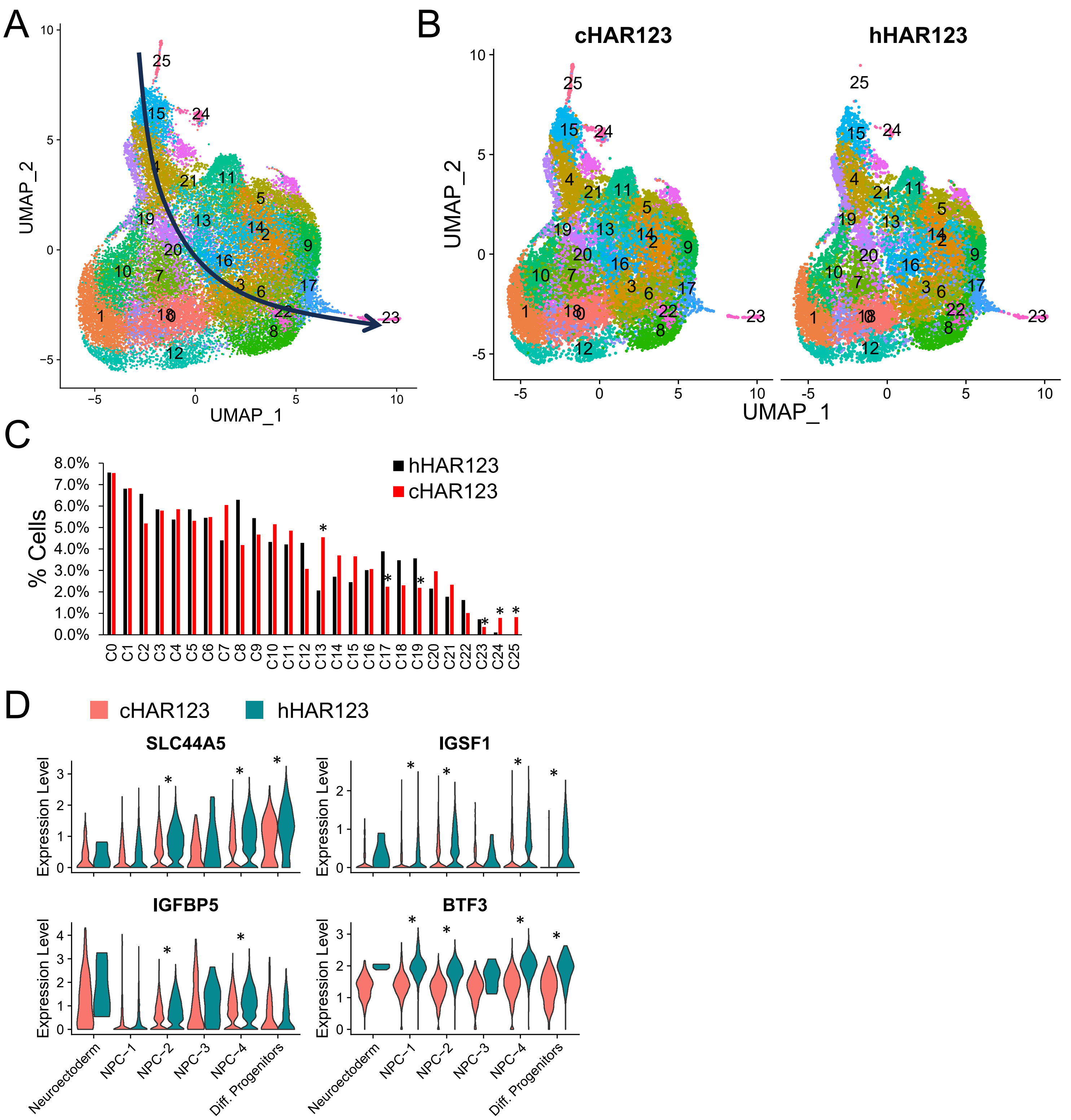

fig. S5. Gene targets and NPC subsets differentially regulated by the human and chimpanzee orthologs of HAR123. (**A**) UMAP plot of hHAR123 and cHAR123 ESCs differentiated into NPCs until just before the rosette selection step. The plot depicts reclustered scRNAseq data from Fig. 2. (**B**) UMAP plots of the same scRNAseq data in panel A, showing the cell contributions from the 2 indicated genotypes. (**C**) The percentage of cells of the indicated genotypes in the subclusters from panel A. *, $p < 0.05$. (**D**) Violin plots showing representative genes differentially expressed between the genotypes shown (from the scRNAseq data described in Fig. 2). *, $p < 0.05$.

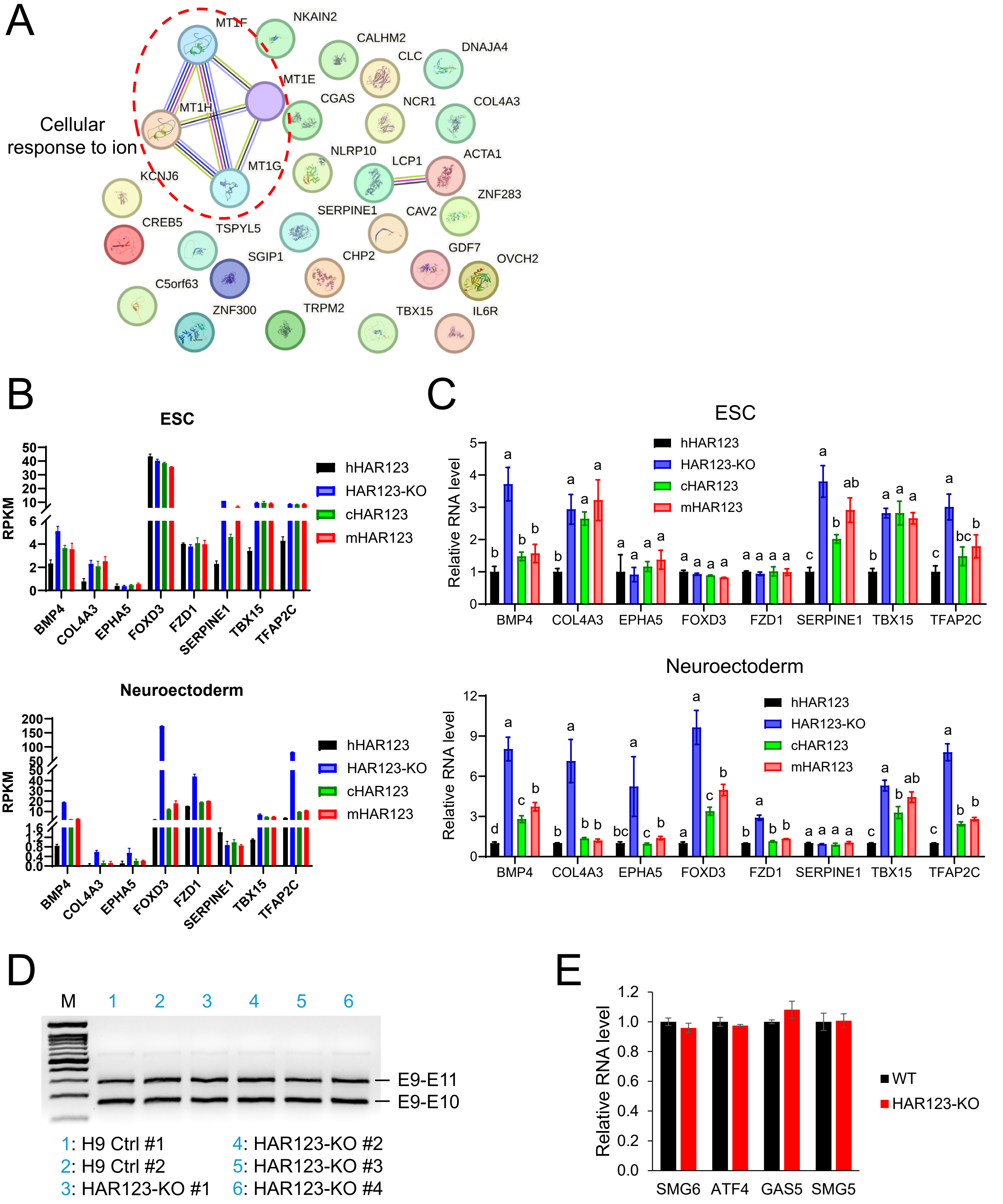
A
Cellular response to ion
MT1F
NKAIN2
CALHM2
CLC
DNAJA4
MT1E
MT1H
CGAS
NCR1
COL4A3
NLRP10
LCP1
ACTA1
MT1G
KCNJ6
SERPINE1
CAV2
ZNF283
CREB5
TSPYL5
SGIP1
CHP2
GDF7
OVCH2
C5orf63
ZNF300
TRPM2
TBX15
IL6R
B
ESC
RPKM
hHAR123
HAR123-KO
cHAR123
mHAR123
BMP4
COL4A3
EPHA5
FOXD3
FZD1
SERPINE1
TBX15
TFAP2C
Neuroectoderm
C
ESC
Relative RNA level
Neuroectoderm
D
M
1
2
3
4
5
6
E9-E11
E9-E10
1: H9 Ctrl #1
2: H9 Ctrl #2
3: HAR123-KO #1
4: HAR123-KO #2
5: HAR123-KO #3
6: HAR123-KO #4
E
Relative RNA level
WT
HAR123-KO
SMG6
ATF4
GAS5
SMG5

fig. S6. hHAR123-regulated genes. (**A**) Genes uniquely regulated by hHAR123 (not cHAR123 or mHAR123) in hESCs, as defined in Fig. 3F. The genes are depicted using functional protein association network analysis. (**B**) Genes differentially regulated in response to different HAR123 orthologs in ESCs and/or neuroectoderm derived from ESCs. The data is from RNAseq analysis (Fig. 3) and is expressed as reads per kilobase of transcript per million reads mapped (RPKM). Shown are randomly-selected genes exhibited different expression patterns. Note that some of these genes are differentially regulated in one cell type but not the other. For example, *EPHA5, FOXD3*, and *FZD1* are differentially regulated in neuroectoderm but not ESCs. Conversely, *SERPINE1* is differentially regulated in ESCs but not neuroectoderm. (**C**) qPCR validation of the genes defined as differentially regulated in panel B. Different letters denote statistically significant differences between groups (p <0.05). n = 3. Data are represented as mean ± SEM. (**D**) RT-PCR analysis of RNA from the indicated cells using a forward primer in exon 9 (E9) and reverse primers in E10 and E11 of SMG6. The "E9-E11" band is the PCR product size expected from the E9/E11 primer pair if there is normal E9/E10/E11 splicing. The "E9-E10" band is the PCR product size expected from the E9/E10 primer pair if there is normal E9/E10 spicing. (**E**) qPCR analysis of *SMG6* mRNA and NMD-target (*ATF4, GAS5,* and *SMG5*) mRNAs in HAR123-KO and control (WT) hESCs. All values are relative to WT cells, which is given a value of "1." n = 3. Data are represented as mean ± SEM.

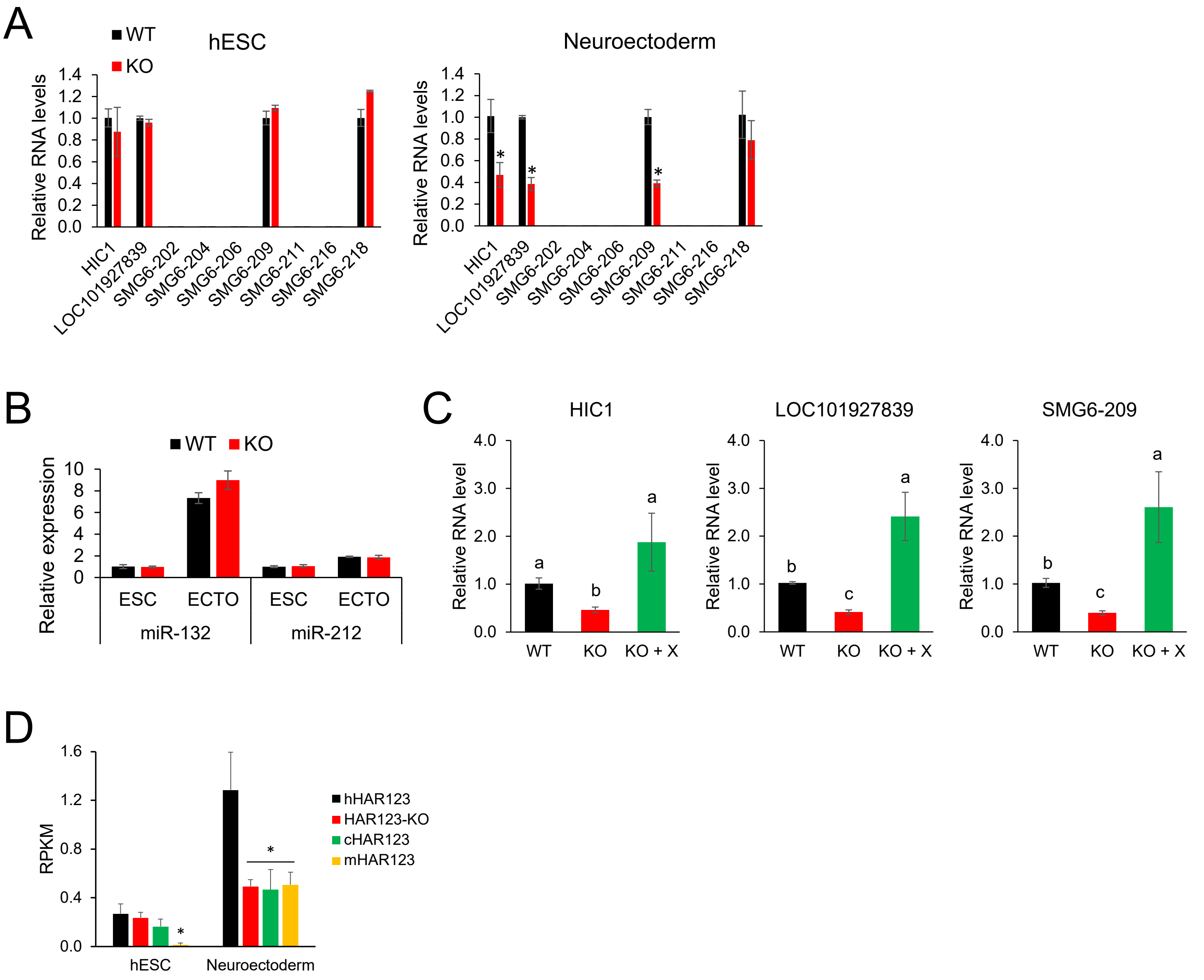

fig. S7. Expression of HAR123 direct target genes. (**A**) qPCR analysis of transcripts originating from promoters within 5 kb of HAR123 interaction sites mapped by HiC analysis (Fig. 4). Their expression was analyzed in undifferentiated or neuroectoderm-differentiated hHAR123 (WT) and HAR123-KO (KO) hESCs. *, p <0.05. KO expression levels are relative to WT, which is set to "1." n = 4. Data are represented as mean ± SEM. (**B**) TaqMan analysis of the indicated microRNAs in the same cells as in panel A. Expression levels are not significantly different between WT and KO. KO expression levels are relative to WT, which is set to "1." n = 4. Data are represented as mean ± SEM. (**C**) qPCR analysis of hHAR123-target gene expression in HAR123-KO hESCs when transiently transfected with an expression vector encoding the same gene (denoted as "X"). Different letters denote statistically significant differences between groups (p <0.05). n = 3. Data are represented as mean ± SEM. (**D**) *HIC1* mRNA expression in ESCs or neuroectoderm of the indicated genotypes, as determined by RNAseq analysis. *, p <0.05.

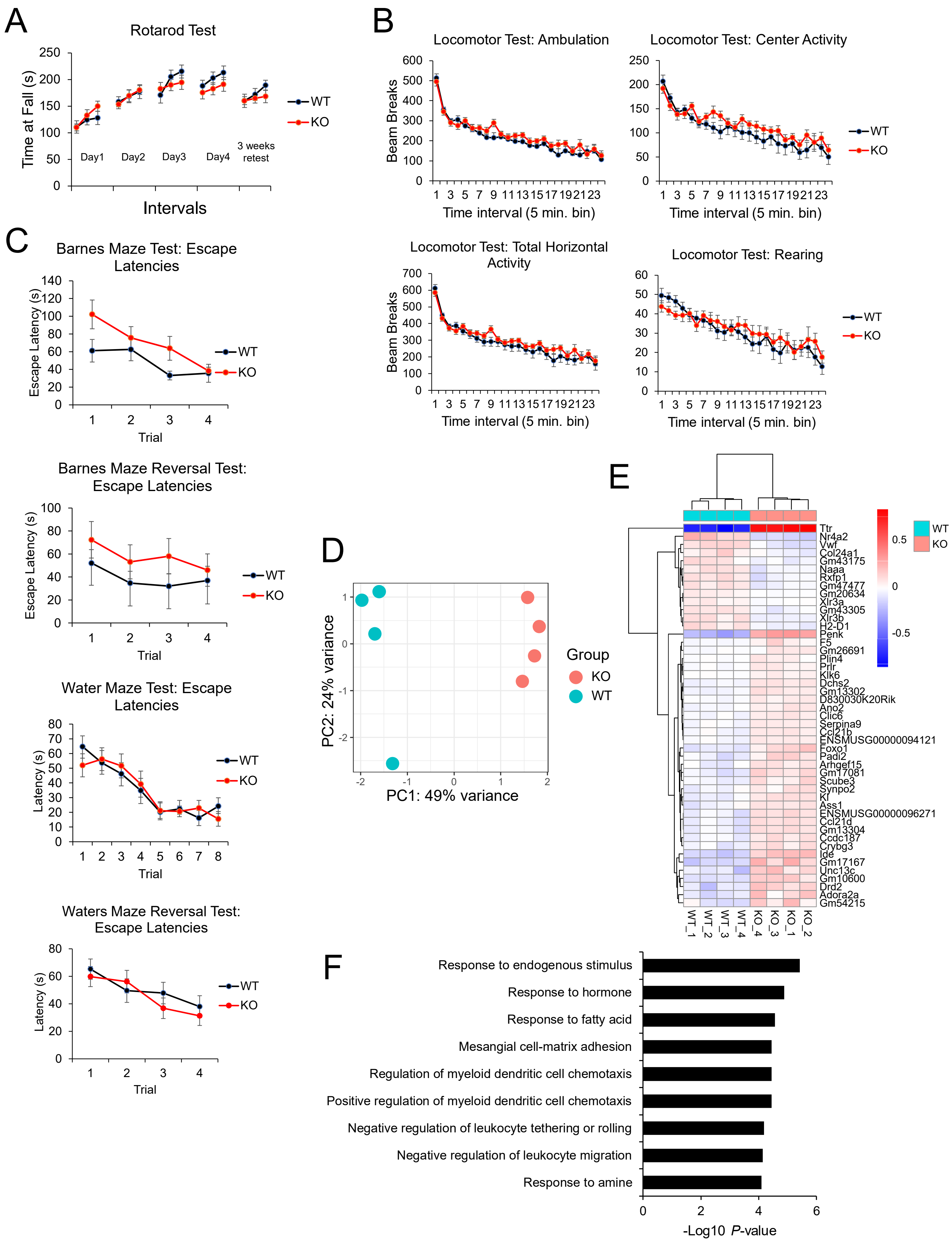

A
Rotarod Test
Time at Fall (s)
Day1
Day2
Day3
Day4
3 weeks retest
Intervals
WT
KO
B
Locomotor Test: Ambulation
Beam Breaks
Time interval (5 min. bin)
Locomotor Test: Center Activity
Locomotor Test: Total Horizontal Activity
Locomotor Test: Rearing
C
Barnes Maze Test: Escape Latencies
Escape Latency (s)
Trial
Barnes Maze Reversal Test: Escape Latencies
Water Maze Test: Escape Latencies
Latency (s)
Waters Maze Reversal Test: Escape Latencies
D
PC2: 24% variance
PC1: 49% variance
Group
KO
WT
E
Ttr
Nr4a2
Vwf
Col24a1
Gm43175
Naaa
Rxfp1
Gm47477
Gm20634
Xlr3a
Gm43305
Xlr3b
H2-D1
Penk
F5
Gm26691
Plin4
Prlr
Klk6
Dchs2
Gm13302
D830030K20Rik
Ano2
Clic6
Serpina9
Ccl21b
ENSMUSG00000094121
Foxo1
Padi2
Arhgef15
Gm17081
Scube3
Synpo2
Kl
Ass1
ENSMUSG00000096271
Ccl21d
Gm13304
Ccdc187
Crybg3
Ide
Gm17167
Unc13c
Gm10600
Drd2
Adora2a
Gm54215
WT_1
WT_2
WT_3
WT_4
KO_4
KO_3
KO_1
KO_2
F
Response to endogenous stimulus
Response to hormone
Response to fatty acid
Mesangial cell-matrix adhesion
Regulation of myeloid dendritic cell chemotaxis
Positive regulation of myeloid dendritic cell chemotaxis
Negative regulation of leukocyte tethering or rolling
Negative regulation of leukocyte migration
Response to amine
-Log10 P-value

fig. S8. Behavioral and molecular analysis of HAR123-KO mice. Panels (**A**) and (**B**) Behavioral analysis performed on adult HAR123-KO and control (WT) littermate mice (n = 16 mice for each genotype). There is no statistical difference between genotypes for any of the tests shown (p >0.05). (**C**) Learning curves for both the Barnes Maze and Water Maze tests shown in Fig. 5, D and E. (**D**) PCA plot of the transcriptomes of frontal cortices from adult HAR123-KO and control (WT) littermate mice (4 samples from each genotype), as defined by RNAseq analysis. (**E**) Heatmap showing the 47 statistically dysregulated genes from the samples described in panel D. (**F**) Statistically enriched biological functions associated with the 47 dysregulated genes shown in panel E.

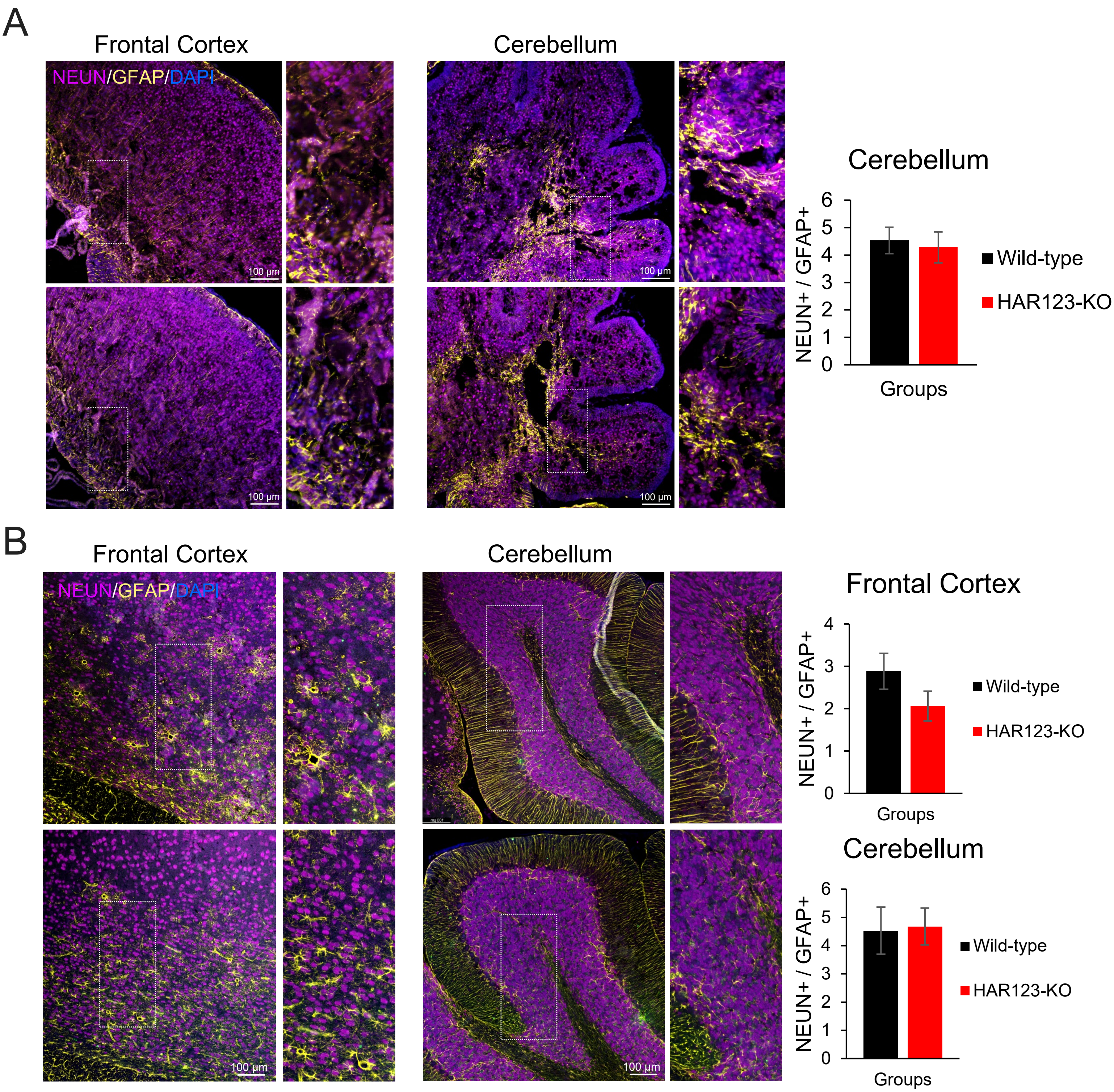
A
Frontal Cortex
Cerebellum
NEUN/GFAP/DAPI
100 µm
Cerebellum
NEUN+ / GFAP+
6
5
4
3
2
1
0
Groups
Wild-type
HAR123-KO
B
Frontal Cortex
Cerebellum
NEUN/GFAP/DAPI
100 µm
Frontal Cortex
NEUN+ / GFAP+
4
3
2
1
0
Groups
Wild-type
HAR123-KO
Cerebellum
NEUN+ / GFAP+
6
5
4
3
2
1
0
Groups
Wild-type
HAR123-KO

fig. S9. Loss of HAR123 does not measurably affect NEUN+/GFAP+ cell ratio in the frontal cortex or cerebellum of postnatal day (P) 7 and P35 mice. (**A**) Left and Middle, immunofluorescence analysis of frontal cortex and cerebellum from P7 mouse brains (sagittal sections) of the indicated genotypes. NEUN marks neurons; GFAP marks glial cells; DAPI marks nuclei. Scale bar, 100 µm. Right, quantification, based on analysis of brain sections from 3 individual mice from each genotype (2 sections per animal). Of note, GFAP+ cells were rare and not well developed in the P7 frontal cortex, so we did not quantify the NEUN+/GFAP+ cell ratio in this region. (**B**) Left and Middle, immunofluorescence analysis of frontal cortex and cerebellum from P35 mouse brains (sagittal sections) of the indicated genotypes, performed and analyzed as in panel A. Right, quantification, based on analysis of brain sections from 2 individual mice from each genotype (3 sections per animal) were analyzed.

Table S5. Differentially expressed genes identified from frontal cortices from HAR123-KO vs littermate wild-type (WT) mice (*q* <0.05, |Log2FC| >0.5)

ID	baseMean	log2FoldChange	pvalue	padj	Symbol
ENSMUSG00000061808	354.7543552	3.237661141	3.12E-220	5.08E-216	Ttr
ENSMUSG00000096039	8.71459083	3.11849391	1.37E-06	0.000293965	D830030K20Rik
ENSMUSG00000002831	6.580315004	2.935362481	8.13E-05	0.009854689	Plin4
ENSMUSG00000026579	11.72569065	2.403755414	1.72E-06	0.00034648	F5
ENSMUSG00000058488	22.564142	1.987459557	9.33E-10	4.90E-07	Kl
ENSMUSG00000050063	7.30042004	1.941341828	0.000562743	0.043853593	Klk6
ENSMUSG00000005268	11.44208721	1.72446774	8.94E-05	0.010475034	Prlr
ENSMUSG00000022949	16.08298804	1.612009549	1.07E-05	0.00173805	Clic6
ENSMUSG00000097911	13.0933959	1.485692194	0.000377208	0.031832055	Gm26691
ENSMUSG00000038115	24.90772076	1.032531969	0.000165308	0.0171489	Ano2
ENSMUSG00000083929	76.79722074	1.03064047	4.64E-11	2.91E-08	Gm10600
ENSMUSG00000058260	22.31466321	1.021398068	0.000413622	0.034520054	Serpina9
ENSMUSG00000090338	59.2173491	0.953387983	8.62E-08	2.86E-05	Gm17081
ENSMUSG00000102692	36.29069119	0.928819946	2.96E-05	0.004388913	Dchs2
ENSMUSG00000096271	54.4437538	0.898577766	1.47E-06	0.000307821	NA
ENSMUSG00000084010	37.10944594	0.897737405	4.54E-05	0.006189524	Gm13302
ENSMUSG00000094065	55.23652099	0.89676529	1.02E-06	0.000223563	Ccl21d
ENSMUSG00000094121	27.28906541	0.886387043	0.000589904	0.045751266	NA
ENSMUSG00000095675	31.68847395	0.87102411	0.000291781	0.025827387	Ccl21b
ENSMUSG00000073878	54.8269141	0.869355072	2.43E-06	0.000482899	Gm13304
ENSMUSG00000038677	46.45383338	0.816802499	5.33E-05	0.007117351	Scube3
ENSMUSG00000045573	649.5862282	0.795580265	7.56E-49	6.16E-45	Penk
ENSMUSG00000091542	191.1831735	0.757102261	1.11E-13	8.64E-11	Gm17167
ENSMUSG00000050315	41.96401106	0.753001074	0.000440786	0.036257994	Synpo2
ENSMUSG00000073879	103.7306145	0.680134994	2.74E-06	0.000512253	Gm54215
ENSMUSG00000052921	54.17436657	0.679878355	0.000215744	0.021167621	Arhgef15
ENSMUSG00000056999	259.0829299	0.668512061	5.53E-16	8.19E-13	Ide
ENSMUSG00000032259	151.343504	0.650750516	6.64E-09	3.18E-06	Drd2
ENSMUSG00000048038	64.33819995	0.621075221	0.000184331	0.019001251	Ccdc187
ENSMUSG00000028927	100.1353257	0.58943548	1.52E-05	0.00235238	Padi2
ENSMUSG00000044167	137.1218264	0.571581095	5.66E-07	0.000139762	Foxo1
ENSMUSG00000076441	68.46544439	0.567427904	0.000447443	0.036620625	Ass1
ENSMUSG00000022723	79.79947454	0.554591694	0.000197329	0.019962139	Crybg3
ENSMUSG00000062151	230.5156186	0.508762303	4.28E-08	1.62E-05	Unc13c
ENSMUSG00000020178	196.5673416	0.506770301	4.79E-07	0.00012202	Adora2a
ENSMUSG00000029413	76.04598877	-0.555049228	0.000255604	0.023519867	Naaa
ENSMUSG00000034009	82.72525477	-0.574757813	9.52E-05	0.010914923	Rxfp1
ENSMUSG00000112905	66.67754614	-0.589459062	0.000277613	0.024980574	Gm47477
ENSMUSG00000105703	59.19650177	-0.620126966	0.000317418	0.027794542	Gm43305
ENSMUSG00000105942	60.71013535	-0.622856852	0.000346047	0.02997909	Gm43175
ENSMUSG00000073411	71.68448661	-0.628408364	6.39E-05	0.008396808	H2-D1

ENSMUSG00000028197	45.40006766	-0.788983348	0.000136848	0.014663453	Col24a1
ENSMUSG00000026826	167.0159919	-0.811505146	2.28E-14	1.96E-11	Nr4a2
ENSMUSG00000001930	70.59240962	-0.899922235	2.69E-08	1.09E-05	Vwf
ENSMUSG00000057836	6.878003616	-2.393780034	0.000164917	0.0171489	Xlr3a
ENSMUSG00000070392	7.696531793	-2.399901867	4.56E-05	0.006189524	Gm20634
ENSMUSG00000073125	16.11922643	-3.070165793	4.92E-11	2.97E-08	Xlr3b

Table S6. Primers used in this study

ID	forward	reverse
SOX17	CTTTCATGGTGTGGGCTAAGG	CTCTGCCTCCTCCACGAAG
TBXT	CAGCAAAGTCAAGCTCACCAAC	GGAAGCAGTGGCTGGTGAT
MIXL1	CAAGCGCACGTCTTTCAGC	CACTCTGACGCCGAGACTTG
HAND1	CACCAGCTACATCGCCTACC	GTGCGTCCTTTAATCCTCTTCTC
SOX2	CACATGAACGGCTGGAGCAAC	GCTGGTCATGGAGTTGTACT
PAX6	GCTGGACAATCAAAACGTGTCC	GGCACTCCCGCTTATACTGG
FABP7	GTCTGTTGTTAGCCTGGATGG	CGAACAGCAACCACATCACC
GAS5	TTGGCACACAGGCATTAGAC	CCATACCCAAGCAAGTCATCC
ATF4	GGCCAAGCACTTCAAACCTC	CATCCTCCTTGCTGTTGTTGG
non-NMD PTBP2	TTACGCCCCAAAGTCTGTTT	TCCATCAGCCATCTGTATTAG
NMD PTBP2	GAGTCTCAGCTGGTGGCAAT	TGCACATCTCCATAAACACCTC
non-NMD HNRNPL	CAATCTCAGTGGACAAGGTG	CCTCCATATTCTGCGGGGTGA
NMD HNRNPL	GGTCGCAGTGTATGTTTGATG	GGCGTTTGTTGGGGTTGCT
non-NMD TRA2B	GAGGTTGGCAGCTTCGATTG	AAGCAGAACGGGATTCCC
NMD TRA2B	TGGAATCAGAAAGCACTACGC	GGAATCTTCCTTGGAGCGAGA
SMG6-206	ATCACACCTGCACTTGGAC	GCAGTAACACCTGGAACTC
SMG6-209	CCTGTAATCCCAGCACTTTG	GCAGTAACACCTGGAACTC
LOC101927839	CAACCGAGAGGAGATCAAGG	TGTTCAGTGCCACCCTTG
HIC1	GACCTGTCCAAGAAGAGCC	GCGAAATGGGTCGGAAGG
SMG6-204	CCAGTTCTTAGGTGGATGGAG	CTGGGAGTTGTGTACTGC
SMG6-218	TGTCTGGAAAGATGACAGGAC	CGACCATACATCCACAGCAAC
SMG6-202	GGACGAGAATGAGAGCTGAAC	GCTGGGAGTTGTGTACTGC
SMG6-211	CTCGTGGAGCTGTTGAGAAC	CTGAGCTTTGGCGGACTC
SOX1	CAACCAGGACCGGGTCAAAC	CCTCGGACATGACCTTCCAC
EOMES	AAGGGGAGAGTTTCATCATCCC	GGCGCAAGAAGAGGATGAAATAG
P75	GTATTCCGACGAGGCCAACC	CAGGGATCTCCTCGCACTC
FOXA2	TTTTAAACTGCCATGCACTCG	TTCATGTTGCTCACGGAGG
SMG6	GCTGGCAGTGTATACGAGGAG	AGGGCTCAGGTCAAATTCCTC
NANOG	AGAGGTCTCGTATTTGCTGCAT	AAACACTCGGTGAAATCAGGGT
SOX2	CACATGAACGGCTGGAGCAAC	GCTGGTCATGGAGTTGTACT
POU5F1	TGGGCTCGAGAAGGATGTG	GCATAGTCGCTGCTTGATCG
RBFOX1	GTTCCCGAGCACACATTAAACC	GTGCTGCGTCATCTGTCTG
WLS	CAAATGCACCTTCACATCTCC	CTCCCCAATTCCCACATTGA

CDH18	TCAATGACAATCCACCCGAAC	CAGGCAGGCGTTCATCAAG
PAX3	GAGACTGGCTCCATACGTC	GATGCGGCTGATGGAACT
FZD5	CAGTTCAACCACGACACGC	GGTAGTCGGGCAGACAGATG
SIX3	CACTCCCACACAAGTAGGC	GGAGGTTACCGAGAGGATGG
FOXG1	CTCACGCTCAACGGCATC	CGAAGCACTTGTTGAGGGAC
SMG6_exon9_F	CTCAGTTTTCTCCATGCCC	
SMG6_exon10_R	GCAAACATATTGATGGTCATAAGC	
SMG6_exon11_R	TTAAGTAAGCAGGTGCAGCG	
FOXD3	GCACTCAAACCCTCTTCCC	GTCGCTGTCCTTCTCTTCC
BMP4	AAGAGCAGATCCACAGCAC	CTCGTTCTCAGGGATGCTG
SERPINE1	TTCCACAAATCAGACGGCAG	CATAAGGGGCAGCAATGAAC
COL4A3	GAAAGGGCAACAAGGCAG	CCCATTTCACCTTGATGACC
TFAP2C	GAAGTTGGACAAGATTGGG	GGCTTCACAGACATAGGC
TBX15	GGATGAGACAGGTGGTCAG	GCTTAGTGGGTGAAAGGTC
EPHA5	TGTGACCGATGAACCTCC	CTGGATGTGAGGTGAGGC
FZD1	AACAGCAAACAAGGGGAG	GCCTGCGAAAGAGAGTTG

Other Supplementary Materials

Table S1. scRNAseq analysis of hESCs differentiated following a standard NPC generation protocol (*20*).
(**Sheet 1**) Significantly enriched genes in each cluster identified by scRNAseq analysis
(**Sheet 2**) Pair-wise comparisons to identify genes differentially expressed in each NPC subsets from scRNAseq analysis (HAR123-KO *vs*. hHAR123)
(**Sheet 3**) The identification of genes differentially expressed in each cell cluster from scRNAseq analysis (cHAR123 *vs*. hHAR123)

Table S2. RNAseq analysis of HAR123-KO, hHAR123, cHAR123, and mHAR123 ESCs and neuroectoderm.
(**Sheet 1**) Differentially expressed genes identified from HAR123-KO *vs*. WT (hHAR123) hESCs (q <0.01, |Log2FC| >1)
(**Sheet 2**) Differentially expressed genes identified from HAR123-KO *vs*. WT (hHAR123) human neuroectodermal cells (q <0.01, |Log2FC| >1)
(**Sheet 3**) Differentially expressed genes identified from cHAR123-KI *vs*. WT (hHAR123) hESCs (q <0.01, |Log2FC| >1)
(**Sheet 4**) Differentially expressed genes identified from cHAR123-KI *vs*. WT (hHAR123) human neuroectodermal cells (q <0.01, |Log2FC| >1)
(**Sheet 5**) Differentially expressed genes identified from mHAR123-KI *vs*. WT (hHAR123) hESCs (q <0.01, |Log2FC| >1)
(**Sheet 6**) Differentially expressed genes identified from mHAR123-KI *vs*. WT (hHAR123) human neuroectodermal cells (q <0.01, |Log2FC| >1)

Table S3. Hi-C analysis of HAR123-KO, hHAR123, cHAR123, and mHAR123 ESCs and neuroectoderm.
(**Sheet 1**) Statistically-significant HAR123-interacting sites identified by Hi-C analysis of hESCs (p <1e-10)

(**Sheet 2**) Statistically-significant HAR123-interacting sites identified by Hi-C analysis of human neuroectodermal cells (p <1e-10)

Table S4. Behavioral assay of HAR123-KO and control littermate mice.
(**Sheet 1**) Elevated Plus Maze Test Raw Data
(**Sheet 2**) Locomotor Activity Test Raw Data
(**Sheet 3**) Optomotor Test Raw Data
(**Sheet 4**) Y Maze Test Raw Data
(**Sheet 5**) Open Field Test Raw Data
(**Sheet 6**) Novel Object Recognition Test Raw Data
(**Sheet 7**) Rotarod Test Raw Data
(**Sheet 8**) Barnes Maze Test Raw Data
(**Sheet 9**) Barnes Maze Reversal Test Raw Data
(**Sheet 10**) Water Maze Test Raw Data
(**Sheet 11**) Water Maze Reversal Test Raw Data
